# Peptoids advance multidisciplinary research and undergraduate education in parallel: Sequence effects on conformation and lipid interactions

**DOI:** 10.1002/bip.23256

**Published:** 2019-01-11

**Authors:** Christian J. Jimenez, Jiacheng Tan, Kalli M. Dowell, Gillian E. Gadbois, Cameron A. Read, Nicole Burgess, Jesus E. Cervantes, Shannon Chan, Anmol Jandaur, Tara Karanik, Jaenic J. Lee, Mikaela C. Ley, Molly McGeehan, Ann McMonigal, Kira L. Palazzo, Samantha A. Parker, Andre Payman, Maritza Soria, Lauren Verheyden, Vivian T. Vo, Jennifer Yin, Anna L. Calkins, Amelia A. Fuller, Grace Y. Stokes

**Affiliations:** ^1^ Department of Chemistry & Biochemistry Santa Clara University Santa Clara California U.S.A.

**Keywords:** chemistry education, fluorescence spectroscopy, sequence‐structure relationships, peptidomimetics, phospholipid membranes

## Abstract

Peptoids are versatile peptidomimetic molecules with wide‐ranging applications from drug discovery to materials science. An understanding of peptoid sequence features that contribute to both their three‐dimensional structures and their interactions with lipids will expand functions of peptoids in varied fields. Furthermore, these topics capture the enthusiasm of undergraduate students who prepare and study diverse peptoids in laboratory coursework and/or in faculty led research. Here, we present the synthesis and study of 21 peptoids with varied functionality, including 19 tripeptoids and 2 longer oligomers. We observed differences in fluorescence spectral features for 10 of the tripeptoids that correlated with peptoid flexibility and relative positioning of chromophores. Interactions of representative peptoids with sonicated glycerophospholipid vesicles were also evaluated using fluorescence spectroscopy. We observed evidence of conformational changes effected by lipids for select peptoids. We also summarize our experiences engaging students in peptoid‐based projects to advance both research and undergraduate educational objectives in parallel.

## 1 INTRODUCTION

Among the many available peptidomimetic molecules, peptoids (*N*‐substituted glycine oligomers) have emerged as an attractive template scaffold for the development of functional molecules compatible with biological environments. Many peptoids, including large combinatorial libraries, have been prepared by the robust sub‐monomer method.^1^ Residue diversity originates from the wide array of commercial or synthetic primary amines available for site‐specific incorporation of side chain functionality. An additional advantage for use of peptoids in biological systems is their resistance to proteolytic degradation.^2^ Researchers have explored peptoids' utility as putative therapeutics.^3^, ^4^ Specific targets have included development of peptoids as antimicrobial agents^5^, ^6^, ^7^, ^8^ and as ligands to a variety of proteins, including vascular endothelial growth factor receptors,^9^ orexin receptors,^10^ and ubiquitin receptors,^11^ to name a few. In parallel, peptoids have shown promise as biocompatible metal‐chelating agents^12^, ^13^ or pH sensors.^14^, ^15^ To expand the applications of peptoids in biological environments, there is an ongoing need to understand the contributions of peptoid sequence both to molecular conformation and to interactions with lipid plasma membranes (PMs).

Peptoids' three‐dimensional structures are an important consideration for their putative function. Peptoid flexibility mediates interaction with target biomolecules, and a number of studies have explored strategies to enhance peptoid affinity for proteins through conformational constraint.^16^, ^17^ In addition, peptoid conformation is important for their function in the detection of metals^12^, ^18^ or in energy transfer,^19^ for example. Recently, we have begun to explore conformation in peptoids that comprise naphthalene‐functionalized residues, enabling study of peptoid structure using fluorescence spectroscopy.^15^ In particular, peptoids that bear naphthalene‐functionalized residues can exhibit excited state dimer (excimer) fluorescence. Importantly, this spectral feature correlates with peptoid structural information because excimers form when chromophores are situated in close proximity (<4 Å).^20^ Excimer emission of naphthalene has also been used as a probe of molecular conformation in other water‐soluble systems. This sensitive and straightforward fluorescence readout has been exploited in pH‐sensing^21^ and metal‐sensing^22^ applications, suggesting that these functions may also be accessible to excimer‐forming peptoids.

A second consideration that will influence peptoid performance in biological environments is their interaction with cellular PMs. The main structural feature of the PM is a lipid bilayer composed mostly of glycerophospholipids. Quantification of drug concentrations partitioned between the aqueous phase and the PM is a critical measurement to evaluate bioavailability and toxicity in the development of new pharmaceuticals.^23^ In addition, the efficacy of antimicrobial peptoids depends on their selective interactions with microbial PMs.^24^ Although peptoids have been shown to have higher cell permeability than peptides,^25^ there are few studies of peptoid‐PM interactions.^5^, ^26^ Advancing peptoid‐based therapeutics^3^ and antimicrobial coatings^5^, ^27^, ^28^ will require new studies that explore relationships between peptoid sequence and structural features and their interactions with PMs.

**Figure 1:**
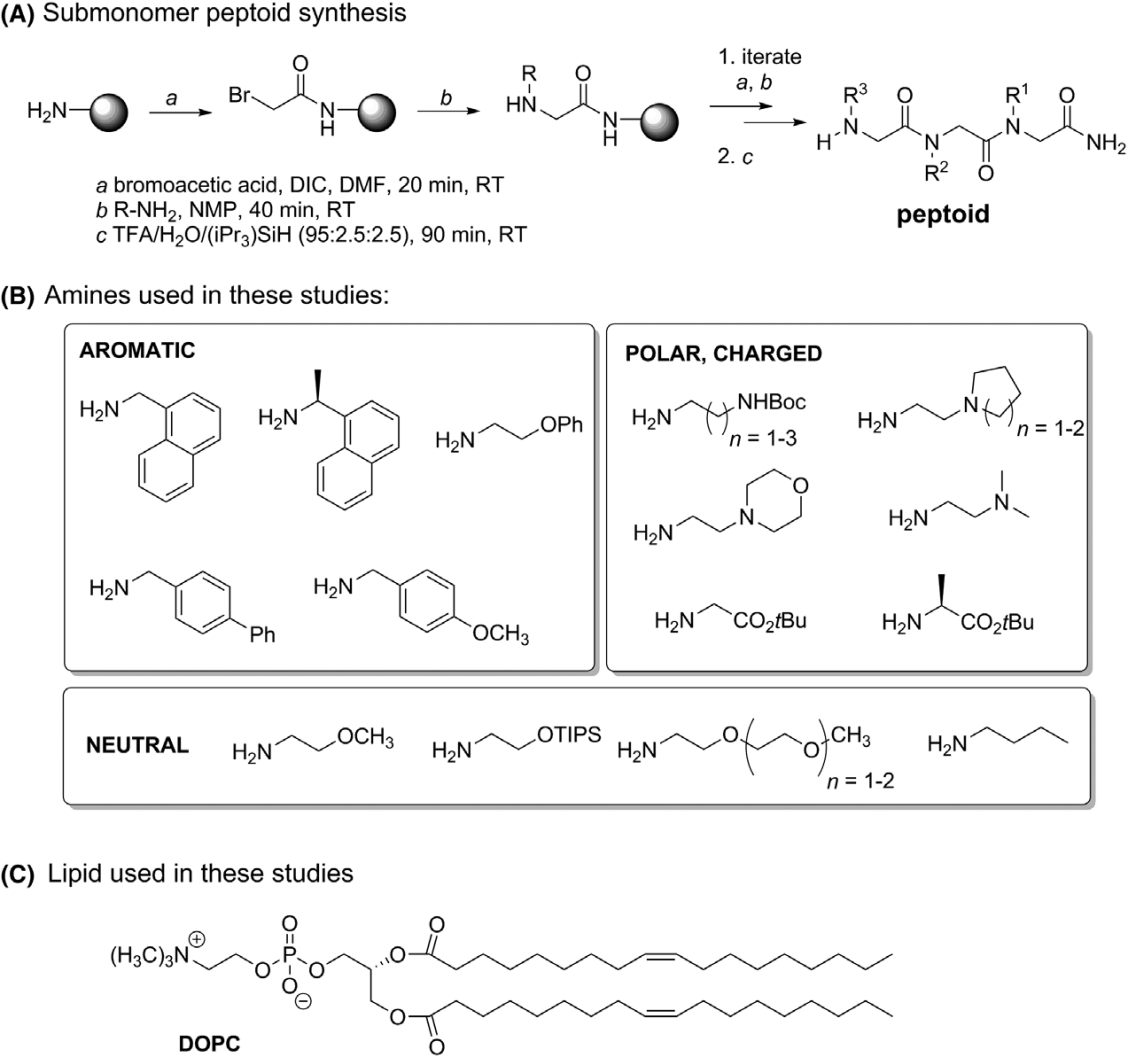
Structures of molecules used in these studies. A, Sub‐monomer synthesis method for preparation of peptoids. B, Amines with varied functionality were used to install side chain functionality sequence‐specifically in an array of 19 tripeptoids and 2 longer peptoid sequences. C, Structure of 1,2‐dioleoyl‐*sn*‐glycero‐3‐phosphocholine (DOPC) lipid used to prepare small unilamellar vesicles as PM mimics

**Figure 2:**
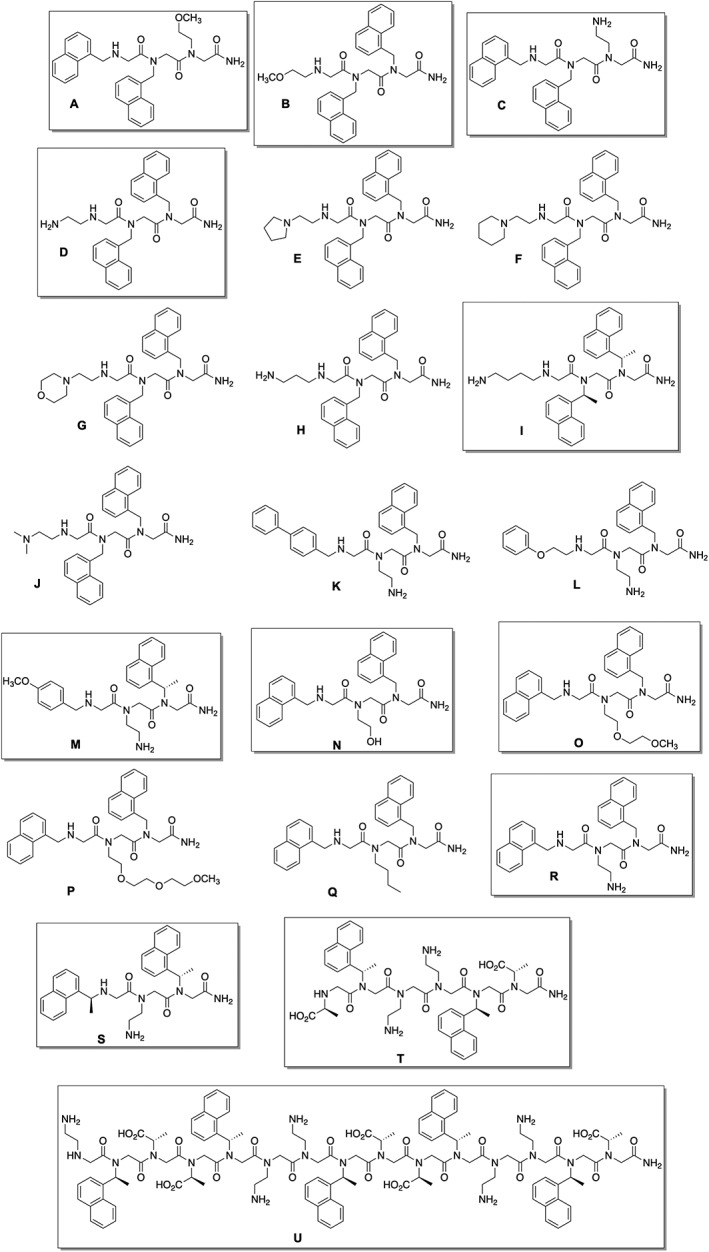
Structures of all peptoids prepared in this work. Boxed structures were studied by fluorescence spectroscopy as detailed in the text

We address the scientific challenges of studying peptoid conformation and peptoid‐PM interactions while simultaneously meeting the need to teach undergraduate students synthetic and analytical research skills. Peptoid research is a rich platform to advance undergraduate learning. Because the applications of peptoids extend to a range of scientific disciplines, students with widely varied interests can find ways to engage with peptoid topics. Importantly, syntheses and spectroscopic studies of peptoids have historically used many approachable techniques, a critical consideration for students with nascent laboratory skills. In this report, undergraduates executed all of the experimental work (Figure 1): they prepared an array of 21 diversely functionalized peptoids (**A‐U**, Figure 2), then evaluated the structures and lipid interactions of 10 representative examples using fluorescence emission spectroscopy in the absence and presence of small unilamellar vesicles (SUVs) composed of 1,2‐dioleoyl‐*sn*‐glycero‐3‐phosphocholine (DOPC) as model PMs.

Undergraduate participation in research activities has been classified as a high‐impact educational practice and is endorsed by such agencies as the President's Council of Advisors on Science and Technology (PCAST)^29^ and the American Chemical Society Committee on Professional Training (ACS‐CPT).^30^ Their experiences in research projects have been shown to advance undergraduate learning and to build students' resilience and confidence.^31^, ^32^ Including research activities in courses (i.e., developing course‐based undergraduate research experiences, CUREs) engages a large number of students in meaningful projects while simultaneously advancing scientific research objectives.^33^, ^34^ Recent publications highlight the contributions of many student researchers enrolled in CUREs to scientific discoveries.^35^, ^36^ Here, we adapt previously reported curricular experiments that engage students in peptoid synthesis^33^, ^37^, ^38^ to include a CURE component—the preparation of novel peptoids for study of their structures and their interactions with model PMs by students enrolled in an introductory organic chemistry laboratory course. In our own experience with this CURE and related work,^39^ students are highly motivated when their work has an immediate application such as this.

Peptoid synthesis experiments are an excellent match with undergraduate organic chemistry laboratory courses. We have implemented a 5‐week peptoid synthesis experiment module in an introductory chemistry laboratory course. The activities that we have carried out are similar to those previously described in the chemical education literature^37^, ^38^, ^39^ (Table 1, refer to detailed student instructions and instructor materials in the Supporting Information). The introduction of peptoids offers an opportunity to engage students with diverse interests; we assigned students to present brief literature reports that summarized the applications of peptoids across multiple sub‐disciplines to situate their hands‐on work in the broader scientific discipline. In addition, peptoid synthesis reactions contextualize concepts addressed in typical introductory organic chemistry lecture courses, including acylation and nucleophilic substitution reactions. The techniques and mechanisms also parallel those typically used for chemical synthesis of peptides.

**Table 1:** Timeline for laboratory activities in tripeptoid synthesis experiments

Week	Laboratory lecture topics	Student presentation topics	Laboratory technique demonstrations	Student laboratory tasks
1	Peptoids and their utility Sub‐monomer peptoid synthesis Solid‐phase synthesis Combinatorial chemistry Protecting groups Introduction to syringe		Synthesis vessel usage	Swell resin Deprotect resin Identify amines for use, do calculations for preparation of at least one amine solution Draw target product in ChemDraw, generate molecular weight
2	Understanding peptoid‐lipid interactions using second harmonic generation spectroscopy	Mechanism: Fmoc removal Peptoid applications in the literature	Chloranil test	Bromoacetylation reaction Amine displacement reaction Bromoacetylation reaction Prepare amine solution
3		Mechanisms: bromoacetylation reaction, amine displacement reaction Peptoid applications in the literature		Amine displacement reaction Bromoacetylation reaction Amine displacement reaction
4	Introduction to Liquid chromatography‐mass spectrometry (LC‐MS) instrumentation and techniques	Mechanisms: chloranil test, cleavage from resin, side chain deprotection Peptoid applications in the literature	LC‐MS sample preparation	Cleave tripeptoid from resin Prepare sample for LC‐MS analysis
5	Introduction to LC‐MS data analysis			Analysis of LC‐MS data

In our class, students researched relevant reaction mechanisms and prepared brief presentations of these. Peptoid synthesis also showcases multistep synthesis, another central concept in organic chemistry courses that can be challenging to incorporate into laboratory coursework. In addition to reinforcing lecture topics, peptoid synthesis provides a platform to introduce modern research techniques less commonly seen at the introductory level, such as combinatorial chemistry and solid phase synthesis.^39^, ^40^, ^41^, ^42^, ^43^

We reasoned that fluorescence emission spectroscopy would provide a meaningful, yet efficient and straightforward technique for undergraduate students to examine peptoid structural features. Peptoid structure comparisons using fluorescence spectroscopy (among other techniques) have been carried out by undergraduate researchers in the Fuller and Stokes laboratories, including in the study of two previously reported, naphthalene‐containing peptoids also used in this work, **T** and **U** (Figure 2).^14^, ^44^, ^45^ **T** and **U** both are water‐soluble and have postulated amphiphilic helix structures, but they exhibit different fluorescence emission features in aqueous buffer. **T** has a single emission peak (340 nm), whereas the emission spectrum of **U** has two emission bands, including a broad emission at 392 nm attributed to an excimer. In the studies reported here, we hypothesized that naphthalene‐functionalized residues would again provide useful fluorescent probes to evaluate peptoid conformation, in particular by correlating sequence features with the presence or absence of the excimer emission.

We speculated that we could also survey peptoids' interactions with model PMs by monitoring changes to peptoids' naphthalene fluorescence emission spectra. This experimental strategy offers the advantage of being able to screen a large number of peptoids to find sequences that exhibit specific photophysical properties in the presence and absence of lipid vesicles. Sensors that rely on fluorescence (including excimer emission) are expected to operate in biological systems. These fluorescent sensors will likely need to be compatible with biological membranes. Several studies have evaluated changes to the fluorescence emission spectrum of a small molecule or peptide upon binding to model PMs,^46^, ^47^ although this approach has not been used to study peptoid‐PM interactions. Measurement of interactions between peptoids or related peptidomimetics and model PMs have been made using X‐ray reflectivity,^5^ isothermal calorimetry^26^; these complement cellular studies.^8^, ^24^, ^48^, ^49^, ^50^ Together, these studies identified that sequence, length, chirality, and charge influenced membrane interactions. In addition to its accessibility to undergraduate researchers, fluorescence spectroscopy offers a method that examines directly the chromophore in the presence and absence of lipids. In the current studies, we extracted qualitative comparisons relating peptoid sequence features with spectral changes upon introduction of lipids.

Here, we report both the preparation of 21 water‐soluble peptoids and their fluorescence emission spectra. We monitored the effects of peptoid sequence on the fluorescence emission spectra at neutral and acidic pH and discovered common sequence features that resulted in excimer formation. In addition, we describe changes to the peptoids' fluorescence spectra when model PMs were introduced. In parallel, this work adds to a growing body of research‐pedagogy publications which describe scientific discoveries made and the educational goals achieved in tandem.^51^, ^52^, ^53^, ^54^ We offer perspectives from our own experiences about undergraduate student researchers' participation in all aspects of these projects to advance both research and educational goals.

## 2 MATERIALS AND METHODS

### 2.1 Materials

Reagents for peptoid synthesis were purchased from commercial suppliers and used without further purification. Alanine‐*tert*‐butyl ester hydrochloride and glycine‐*tert*‐butyl ester hydrochloride were purchased from AAPPTec (Louisville, KY). Before use, the ammonium salt was dissolved in a minimal amount of water, and 2.5 M NaOH was added to adjust the solution pH to 10. The neutralized amine was then extracted with CH_2_Cl_2_, and the organic solution was dried over MgSO_4_, then concentrated by rotary evaporation. 2‐((Triisopropylsilyl)oxy)ethan‐1‐amine was prepared according to the reported procedure, and spectroscopic properties were identical to those reported.^55^ 2‐(2‐Methoxyethoxy)ethan‐1‐amine and 2‐(2‐(2‐methoxyethoxy)ethoxy)ethan‐1‐amine were prepared by the laboratory of Ronald Zuckermann according to reported procedures.^48^ Fmoc‐protected Rink amide resin was purchased from EMD Millipore. Solvents were purchased from Fisher Scientific (Hampton, NH) unless otherwise specified. Chloroform (HPLC grade) was purchased from Honeywell Burdick and Jackson (Muskegon, MI) and was dried over 4 Å molecular sieves (Alfa Aesar, Haverhill, MA) before use in the preparation of dried lipids as detailed below. Peptoids **T** and **U** were prepared as previously described.^15^

Before students initiated peptoid synthesis experiments, the instructor stressed the safety precautions related to working with organic chemicals to all students both in the written protocol and also by verbal reminders. In particular, we emphasized to the students that many chemicals are significant health hazards, including solvents used in larger volumes: *N*,*N*‐dimethylformamide (DMF, amine free), CH_2_Cl_2_, *N*‐methyl‐2‐pyrrolidone (NMP). Many of the amines used and other chemicals handled in smaller quantities (including 4‐methylpiperidine, *N*,*N′*‐diisopropylcarbodiimide [DIC], bromoacetic acid) are flammable and/or corrosive. Lastly, we stressed that trifluoroacetic acid (TFA) used in the cleavage of the peptoid from the resin is a very strong acid, and students were closely monitored while using this reagent. Detailed safety information about the full suite of chemicals used is included in the Supporting Information.

### 2.2 Tripeptoid synthesis

Solid‐phase tripeptoid syntheses were executed by undergraduates, most commonly in the introductory organic chemistry laboratory course. Detailed student procedures and instructor materials for implementing peptoid synthesis experiments as laboratory course experiments are included in the Supporting Information. Peptoid syntheses were done in syringes fitted with coarse frits purchased from Torviq (Niles, MI). The syringe plunger was removed to add Fmoc‐Rink amide resin (0.1 mmol) to the syringe barrel, then the plunger was replaced. Reagent solutions or wash solvents were drawn into or pushed out of the resin using the syringe plunger. Syringes were capped during resin swelling and reaction times.

A syringe charged with Fmoc‐Rink amide resin was washed with DMF three times, then allowed to swell for at least 10 minutes in the capped syringe. The DMF was removed from the syringe, and the resin was treated with 2 mL of a 20% 4‐methylpiperidine solution in DMF for 15 minutes. The resin was washed twice with DMF, then equilibrated with another 2 mL of a 20% 4‐methylpiperidine solution in DMF for 15 minutes. The resin was drained, then washed with DMF 10 times. A mixture of 1.7 mL of 1.2 M bromoacetic acid in DMF and 0.4 mL DIC was drawn into the syringe.

The syringe was capped, and the reaction was allowed to proceed for 20 minutes with occasional manual agitation of the syringe. The reaction solution was expelled from the syringe, and the resin was washed with DMF (four times) and NMP (four times). To the syringe was added 2 mL of a 1 M solution of the appropriate amine in NMP. The syringe was capped, then subjected to occasional manual agitation. After 40 minutes, the reaction mixture was expelled from the syringe, and the resin was washed with DMF (eight times). A few resin beads were removed and subjected to the chloranil test to confirm the presence of the secondary amine (blue‐green beads). The bromoacetic acid/DIC and amine displacement reactions were iterated twice more using the appropriate amines to complete the peptoid synthesis. Again, reaction completion at each step was monitored using the chloranil test to assess the presence or absence of a secondary amine. The resin was then washed with CH_2_Cl_2_ five times, then the syringe plunger was removed, and the resin was allowed to dry open to air in the hood for at least 10 minutes. The syringe was capped, clamped upright to a support in the hood, and 5 mL of a solution of 95% TFA/2.5% H_2_O/2.5% triisopropylsilane were added to the resin. After 60 to 90 minutes, the cap was removed, and the filtrate was collected into a clean, tared vial. TFA was removed by evaporation.

### 2.3 Peptoid purification and identification

Crude tripeptoid **O** was purified by column chromatography using a Hypersep Si column (500 mg/3 mL) using 5% methanol in dichloromethane, then 10% methanol in dichloromethane to effect complete elution from the column. The remaining tripeptoids were purified by reverse‐phase high performance liquid chromatography (RP‐HPLC) on an Agilent Technologies 1260 Infinity II system equipped with a Polaris C18‐A column (250 × 10.0 mm, 5 μm) using a 10 minute linear gradient of 30%‐90% methanol (solvent B) in 0.1% aqueous TFA (solvent A) at a flow rate of 4.7 mL/min. Peaks eluted were detected by absorbance at 220 nm. All data were visualized with OpenLab CDS software.

Peptoids were identified by electrospray mass spectrometry in positive‐ion mode using a Thermo LCQ Fleet mass spectrometer. High‐resolution mass spectral data were collected for 10 representative tripeptoids using an Agilent 1260 Infinity II LC with 6230 time of flight mass spectrometer (TOF MS) detector (electrospray ionization, positive ion mode) and were within 5 ppm of expected values. These data are tabulated in the Supporting Information, Table S1. Purified peptoids were lyophilized to white powders.

### 2.4 Buffer preparation

Phosphate‐buffered saline (PBS buffer) was prepared with 50 mM sodium phosphate (Fisher Scientific) and 100 mM sodium chloride (Alfa Aesar) using ultrapure 18 MΩ water. The pH of the PBS buffer was adjusted to 7.4 with sodium hydroxide (VWR, Radnor, PA) or hydrochloric acid (MilliporeSigma, Burlington, MA). PBS buffers were stored at 4 °C. Acetate buffer (pH 5.0) was prepared with 50 mM sodium acetate (MilliporeSigma, Burlington, MA) and 183 mM sodium chloride (VWR, Radnor, PA) to maintain total ionic strength at 215 mM.

### 2.5 Peptoid stock preparation

Peptoid stocks solutions (1‐2 mM) were prepared by diluting lyophilized peptoid into HPLC‐grade methanol. Stock concentrations were determined by UV spectroscopy using experimentally determined extinction coefficients at 266 nm as tabulated in the Supporting Information, Table S2. All peptoids contained naphthalene residues which exhibit strong absorbance at 266 nm. Peptoid solutions for spectroscopic studies were prepared by diluting the methanol stock solution into the appropriate buffer.

### 2.6 SUV preparation

Following standard procedures,^49^ we prepared dried lipid films (2.62 mg/mL) from stock DOPC dissolved in chloroform (25 mg/mL) purchased from Avanti Polar Lipids (Alabaster, AL). The lipids were evaporated under a gentle stream of nitrogen gas (ultra high purity, Matheson) and vacuum dried overnight to remove residual chloroform. Dried lipid aliquots (7.85 mg) were stored at −20 °C. SUVs were prepared by reconstituting dried lipid films with 3 mL PBS buffer (lipid concentration = 3320 mM) followed by vortexing to mix and bath sonication for 20 minutes until solutions were clear. Average diameter and polydispersity of SUVs made by this procedure were determined by dynamic light scattering (90Plus Particle Size Analyzer, Brookhaven Instruments Corp, Holtsville, NY). Sonicated SUVs had a mean diameter of 111.9 ± 2.2 nm, and the polydispersity was 0.269 ± 0.005.

SUVs made in the appropriate buffers were subsequently studied by fluorescence spectroscopy. Fluorescence measurements were complete within 2 hours after sonication to address the potential that SUVs prepared by sonication may not be stable over long periods of time.^51^ Equal volumes of sonicated SUVs and aqueous peptoid solutions were added together, mixed by vortexing, and allowed to equilibrate to final concentrations of 1660 μM lipid and 100 μM tripeptoids. Final concentrations of 4000 μM lipid and 40 μM peptoid were used for longer peptoids **T** and **U**. Experiments with **T** and **U**, which had higher molar absorptivities due to more chromophores, required lower final peptoid concentrations. A higher lipid‐to‐peptoid ratio ensured that each naphthalene on **U**, which was five times longer than a tripeptoid, encountered at least as many lipids as each naphthalene on a tripeptoid. For measurements of peptoid‐lipid mixtures at pH 5.0, SUVs were initially formed by sonication in PBS pH 7.4 until clear, as described above, then diluted into pH 5.0 acetate buffer to the concentrations reported above. This ensured that acidic conditions would not destabilize the lipid bilayers when SUVs were formed during sonication.^52^

### 2.7 Fluorescence spectroscopy

Fluorescence emission spectra of tripeptoids **A**, **B**, **C**, **D**, **I**, **M**, **N**, **O**, **R**, and **S** were acquired on a Molecular Devices SpectraMax i3x plate reader instrument, and experimental setup and data extraction were done with SoftMax Pro 6.5.1. Solutions for analysis were pipetted into black, flat‐bottom Thermo Scientific Microtiter 96‐well plates. Total solution volume in each well was 150 mL, and three wells were filled from each solution. The data reported are the average of these three replicates. Emission spectra were collected from 300 to 500 nm in 1 nm increments. The excitation wavelength was set to 270 nm, and excitation and emission slit widths were set to 9 nm and 15 nm, respectively.

For the fluorescence spectra of **T** and **U**, a Horiba Fluorolog 3 spectrofluorometer was used. Samples were excited at 270 nm, and emission was scanned from 300 to 500 nm in 1 nm increments with excitation and emission slit widths set to 6 nm and 3 nm, respectively. All experiments were conducted in 50 mM phosphate buffer, pH 7.4 with no added NaCl.

## 3 RESULTS AND DISCUSSION

### 3.1 Peptoid design

An array of tripeptoids was designed for preparation by undergraduate students enrolled in an introductory organic chemistry laboratory CURE course (Figure 2). Tripeptoids were chosen because they could be prepared in a reasonable timeframe in instructional laboratories (Table 1). We speculated that small peptoids would likely be more water soluble, enabling their later study by optical methods without observing scattering effects.^53^, ^56^ In addition, in considering the potential applications of peptoids in biological environments, aqueous solubility was important. Lastly, tripeptoids offered three positions to vary functionality, including the essential naphthalene chromophores, to begin to survey the contributions of individual residues to differences in fluorescence spectra. Initial peptoid sequences were designed by faculty to survey a wide variety of photophysical and physicochemical properties. We expect that future iterations of this CURE will incorporate student‐designed molecules informed by the data reported here (*vide infra*). Because we were interested in identifying sequences with excimer emission, most tripeptoids comprised two naphthalene‐functionalized side chains in adjacent or nonadjacent positions. Other appended functionalities were varied to include other aromatic groups (peptoids **K**, **L**, **M**) that might contribute to the optical properties of the molecule and/or moieties that modulated peptoid solubility in water and net charge: neutral polar groups (peptoids **A**, **B**, **N**, **O**, **P**), charged polar groups (peptoids **C**‐**M**, **R**, **S**).

Two sequences prepared included (*S*)‐1‐naphthylethyl side chains in place of the more common 1‐naphthylmethyl side chains (**I**, **M**). Admittedly, the substitution of the (*S*)‐1‐naphthylethyl side chains in **I** and **M** was accidental; students misread the bottle labels and used the “incorrect” amine (a reasonably frequent error of an inexperienced chemist). Nonetheless, this “mistake” provided an opportunity to diversify our sequences. Tripeptoid **I** in particular is a close analog of isomeric tripeptoids **C**, **D**, and **R**. Furthermore, the process of working backwards to validate the unintended sequence and discover the origin of this substitution was an excellent learning experience for the students.

Two additional, longer peptoids were also prepared for study, **T** and **U** (Figure 2). **T** and **U** are both water‐soluble and have putative amphiphilic helix structures. In our previous studies with **T** and **U**, we learned that fluorescence spectra of peptoids with multiple chromophores can change with peptoid conformation. Their inclusion in these studies enabled preliminary investigation of lipid influence on photophysical features of longer peptoids.

### 3.2 Tripeptoid synthesis and educational considerations

Peptoids were prepared on solid support by the sub‐monomer synthesis approach^1^ (Figure 1A) in which each peptoid residue was installed in two synthetic steps. Fmoc‐Rink amide resin was deprotected, then subjected to DIC‐mediated acylation with bromoacetic acid. The bromine was subsequently displaced by reaction with an excess of a primary amine bearing the functionality corresponding to the *C*‐terminal peptoid residue. Alternating treatment of the resin‐bound intermediate with bromoacetic acid/DIC and primary amines was repeated two more times to install site‐specifically each residue. Completion of reactions was monitored by subjecting a few beads to the chloranil test; blue‐green beads indicated the presence of a secondary amine. The tripeptoid was liberated from the solid support with concomitant removal of side chain functionality protecting groups by treatment with strong acid.

In the CURE laboratory course setting, each student was charged with preparing one unique peptoid, and 17 of the peptoids (**A**‐**Q**, Figure 2) were initially prepared in this fashion. Data from liquid chromatography‐mass spectrometry (LC/MS) experiments allowed students to estimate crude purities and verify if their syntheses were successful. Of the 17 tripeptoids prepared in the laboratory course, 13 were successfully prepared in >85% crude purity, two were prepared in lower crude purity, and two were not identified from the students' syntheses. The low‐purity and unsuccessful sequences were subsequently remade successfully by independent undergraduate research students along with peptoids **R**‐**U**. Detailed student procedures and instructor materials used in this course are included in the Supporting Information. The high level of success in tripeptoid preparation by undergraduate students highlighted the robustness of the solid‐phase sub‐monomer synthesis methods in the hands of novice experimentalists. Students who were entirely unfamiliar with the equipment and techniques involved in solid phase synthesis were able to execute these experiments reliably.

Before their use in spectroscopic studies, peptoids were purified by RP‐HPLC, and their identities were confirmed by mass spectrometry. RP‐HPLC purification was carried out outside of the CURE laboratory course setting owing to the time‐consuming nature of this more technically demanding procedure.

### 3.3 Selection of peptoids for fluorescence spectroscopy

From our library of 19 tripeptoids prepared, we selected 10 for further study by fluorescence spectroscopy in the absence and presence of SUVs: **A**, **B**, **C**, **D**, **I**, **M**, **N**, **O**, **R**, and **S**. These were chosen to enable us to correlate tripeptoid sequence features (e.g., residue identity, conformational flexibility, and residue ordering) with spectroscopic observations. Importantly, we selected five peptoids with two adjacent napthalene‐functionalized residues (**A‐D**, **I**), and four that included two naphthalene‐functionalized residues in nonadjacent positions (**N**, **O**, **R**, **S**). Peptoids also included polar groups that were expected to influence peptoid net charge: **A**, **B**, **N**, and **O** all comprise a polar, uncharged side chain, whereas **C**, **D**, **I**, **M**, and **R** included a basic side chain, and **S** contained an acidic side chain. Peptoids **C**, **D**, and **R** were all isomers; comparisons across this series enabled analysis of the effects of specific residue positioning on spectroscopic features. Peptoids **I** and **M** included (*S*)‐1‐naphthylethyl side chains; these are known to favor the *cis* amide conformation strongly.^57^ We expected that **I** and **M** would allow us to investigate the influence of conformational flexibility on fluorescence spectral features and/or lipid interactions.

### 3.4 Fluorescence emission of tripeptoids in buffers at varied pH

**Figure 3:**
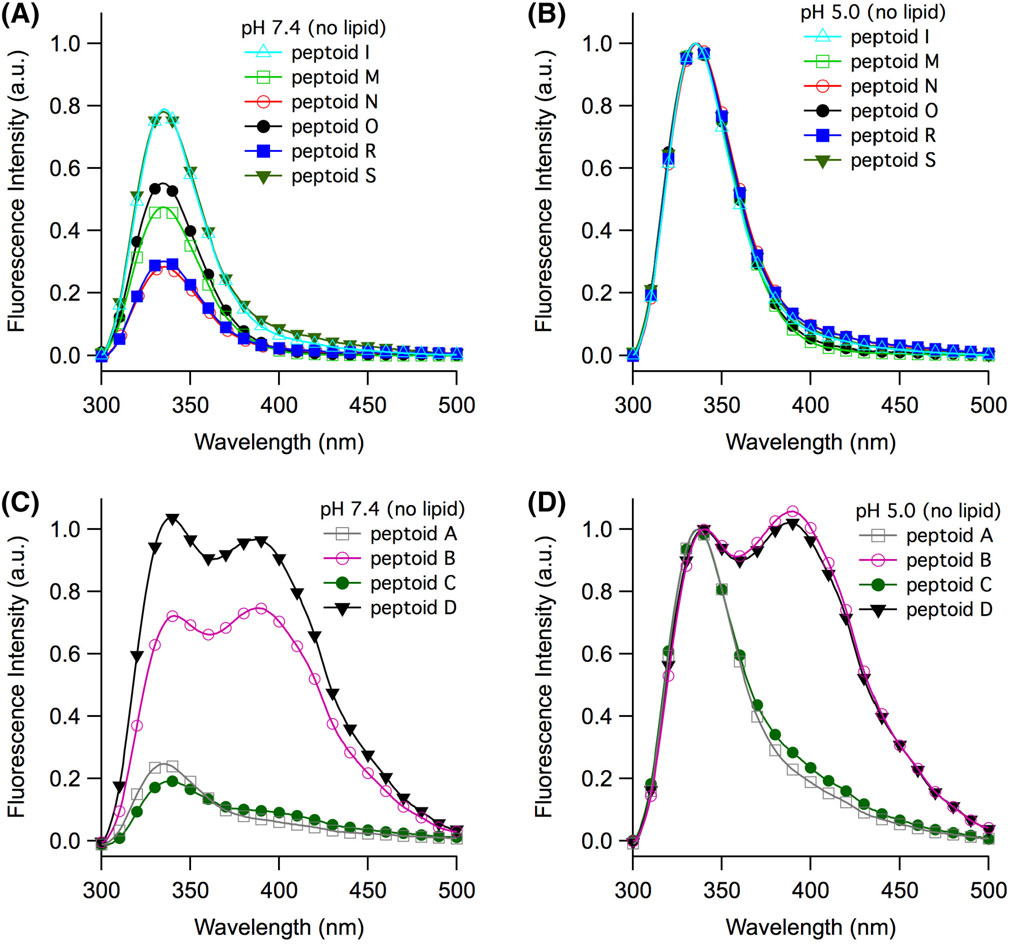
Fluorescence emission spectra of tripeptoids **I**, **M**, **N**, **O**, **R**, and **S** (panels A and B) and **A**, **B**, **C**, and **D** (panels C and D). Experiments were conducted at pH 7.4 (panels A and C) and at pH 5.0 (panels B and D). Dissolved peptoid concentrations were maintained at 100 μM. Data were normalized to the lower wavelength peak for each peptoid sequence at pH 5.0

We collected fluorescence emission spectra for the 10 representative tripeptoids to investigate their possible structural differences. A summary of fluorescence data is shown in Table 2. Spectra were acquired in two different buffers—at pH 7.4 (Figure 3A and C) and at pH 5.0 (Figure 3B and D). We anticipated that pH might influence peptoid conformation and therefore fluorescence emission features; this has been observed in other bichromophore systems.^21^ Human blood is commonly modeled with pH 7.4 PBS buffer, whereas the gastrointestinal environment is commonly modeled at pH 5.0.

**Table 2:** Summary of fluorescence emission observations for tripeptoids

Tripeptoid	*λ*_max_ a (nm)	*I*_5.0_/*I*_7.4_ b (no lipid)	*I*_5.0_/*I*_7.4_ (with lipid)	*I*_lipid_/*I*_buffer_ c (pH 5.0)	*I*_lipid_/*I*_buffer_ (pH 7.4)
**A**	335	4.04	6.88	2.68	1.58
**B**	341	1.38	1.12	1.39	1.72
	**387**	**1.42**	**0.98**	**0.71**	**1.02**
**C**	338	5.21	6.70	2.34	1.81
**D**	339	0.96	0.90	1.71	1.84
	**389**	**1.07**	**0.82**	**0.45**	**0.98**
**I**	335	1.27	1.02	0.97	1.21
**M**	335	2.10	2.00	1.26	1.32
**N**	335	3.53	3.62	1.83	1.78
**O**	335	1.81	1.57	1.39	1.61
**R**	335	3.31	4.42	2.13	1.59
**S**	335	1.28	1.17	1.25	1.36

a *λ*_max_ emission in pH 7.4 buffer in the absence of small unilamellar vesicles (SUVs).

b Ratio of fluorescence emission intensity at *λ*_max_ for solutions of peptoid at pH 5.0 and pH 7.4.

c Ratio of fluorescence emission intensity at *λ*_max_ for solutions of peptoid in the presence and absence of 16.6‐fold excess of SUVs. Data for excimer peaks are given in bold.

Fluorescence emission spectra for tripeptoids **I**, **M**, **N**, **O**, **R**, and **S** exhibited a single peak with *λ*_max_ at 335 nm at pH 7.4 (Figure 3A). This is a typical spectral feature for emission of naphthalene‐comprising molecules in water. Upon decreasing the solution pH to 5.0, the *λ*_max_ did not shift, but emission intensities increased (*I*_5.0_/*I*_7.4_ > 1, Table 2, Figure 3B). Other researchers have shown that neutral amines quench naphthalene fluorescence either intermolecularly or intramolecularly.^21^ At lower pH, we likewise observed greater fluorescence efficiency because the peptoids were more positively charged.

The spectra of **A**‐**D** had notably different features from those of the other peptoids studied (Figure 3C and D). At pH 7.4, the emission spectra of **A** and **C** exhibited a shoulder around 390 nm. Most strikingly, the emission spectra of **B** and **D** each had two peaks: for **B**, these were at 341 nm and 387 nm, and for **D**, these were at 339 nm and 389 nm. The broad, red‐shifted peaks in the spectra of **B** and **D** were characteristic of naphthalene excimer emission and suggested that the two naphthalene chromophores were positioned close to one another (usually <4 Å).^20^ When solution pH was decreased to 5.0, the intensity of the excimer (higher wavelength) peak relative to the monomer emission peak increased slightly for **B** and **D** (Figure 3D). In contrast, the excimer‐to‐monomer intensity ratio was slightly lower for **A** and **C** at lower pH.

Intensity changes to fluorescence spectra in response to pH (*I*_5.0_/*I*_7.4_, Table 2) were most influenced by the proximity of the naphthalene to the ionizable secondary amine at the *N*‐terminus of the tripeptoid. Peptoids **C**, **A**, **N**, and **R** displayed the greatest *I*_5.0_/*I*_7.4_; these had naphthylmethyl side chains in the *N*‐terminal position, closest to the secondary amine that could most efficiently quench fluorescence. In contrast, when the two naphthalenes were farther from the *N*‐terminus, spectral intensities were less sensitive to pH changes (e.g., **B**, **D**, and **I**).

The excimer emission observed for **A**‐**D** was correlated with specific sequence features of these tripeptoids. In all of these, the two residues bearing 1‐naphthylmethyl side chains were adjacent; in **A** and **C**, they were on the *N*‐terminus of the molecule, whereas in **B** and **D**, they were on the *C*‐terminus. The adjacency of these two residues to one another enabled the tripeptoids to adopt a conformation in which the two naphthalenes were close enough in space to form an intramolecular excimer. For **B** and **D**, we observed a higher ratio of excimer/monomer emission peak intensities at lower pH. We speculated that this could originate in either (or both) of two ways: first, the greater positive charge on the molecule at lower pH strengthened intramolecular hydrophobically driven association of the naphthalenes, limiting their conformational freedom. Alternately (or in parallel), the conformational ensembles were similar at both pHs, but excimer emission in **B** and **D** was quenched more efficiently at higher pH.

Intramolecular excimers were observed in analogous water‐soluble bichromorphoric molecules.^21^, ^22^ Our data suggested that the conformation that enabled this excimer was most favorable when the two naphthylmethyl groups were attached to the two *C*‐terminal residues. When the two naphthylmethyl residues were nonadjacent, the conformation that enabled excimer emission was not favored (e.g., **N**, **O**, **R**, **S**). Given the relatively low concentrations at which these studies were carried out (100 μM), we expected that intermolecular association of chromophores was unlikely. To confirm this, we noted that the ratio of excimer to monomer emission intensities did not change for peptoid **D** at lower concentrations (Supporting Information Figure S1).

Interestingly, the emission spectra of **I** (Figure 3A and B), which was structurally quite similar to **D**, did not include excimer emission. Although both **D** and **I** comprised two naphthalene‐functionalized *C*‐terminal residues, the (*S*)‐1‐naphthylethyl side chains in **I** conferred less conformational flexibility than the 1‐naphthylmethyl side chains in **D**. We inferred that the enhanced conformational mobility of **D** was necessary for the molecule to adopt a conformation that enabled intramolecular excimer formation. In analogous studies, rigid scaffolds that bear several naphthalene chromophores did not exhibit excimer fluorescence; indeed, the absence of the excimer peak has been used as an indicator of structural rigidity.^58^, ^59^

### 3.5 Fluorescence emission of peptoids in the presence of SUVs

To evaluate peptoid‐lipid interactions, we acquired fluorescence emission spectra of 10 representative tripeptoids in the presence of 16.6‐fold excess SUVs (Supporting Information Figure S1, Table 2). We chose to use SUVs as model PMs because of their optical compatibility and because they are straightforward to prepare by sonication.^60^ SUVs are a common model PM to probe fluorophore‐lipid interactions.^46^, ^61^, ^62^, ^63^ Because >50% of the glycerophospholipids found in eukaryotic PMs are zwitterionic and in the fluid phase,^64^ we chose to use SUVs composed of DOPC (structure shown in Figure 1). Introduction of SUVs into solutions of aqueous peptoids resulted in highly complex mixtures for analysis; our fluorescence spectra convoluted lipid‐associated peptoids and peptoids partitioned in the aqueous phase. Changes in fluorescence emission intensities of molecules in the presence of lipids may be attributed to a number of factors. Localization into the membranes situates chromophores in a less polar environment that may limit some collisional quenching. Alternately, membrane introduction may induce a conformational change, limiting non‐radiative fluorescence quenching pathways. Using only steady‐state fluorescence emission spectroscopy, we were unable to quantify adsorbed chromophores, elucidate a binding mechanism, or determine the orientation or location of bound peptoids (e.g., at the periphery, inside the SUV, or spanning the membrane). Despite these limitations, comparisons of spectra collected in the presence and absence of SUVs highlighted the structural motifs that contributed to changes in fluorescence emission. This information provided valuable insight into how a putative sensor peptoid that relies on a specific conformation would behave when interacting with PMs.

**Figure 4:**
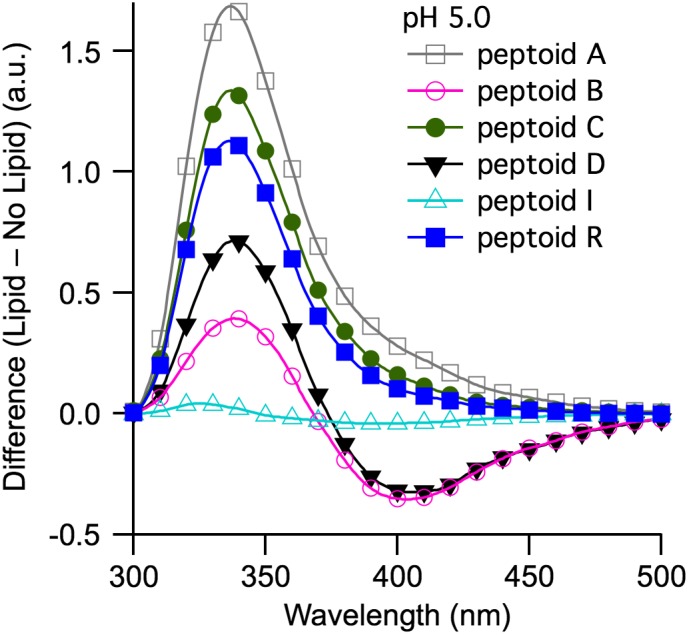
Difference in fluorescence emission intensities in the presence minus in the absence of SUVs composed of DOPC for tripeptoids **A**, **B**, **C**, **D**, **I**, and **R**. Experiments were conducted at pH 5.0. Dissolved peptoid concentrations were maintained at 100 μM. A lipid‐to‐peptoid molar ratio of 16.6:1 was used for all peptoid sequences

We focused first on comparisons of spectral features between only 6 of the 10 peptoids studied. In particular, we were interested in evaluating changes upon SUV introduction to the spectral features in **A**‐**D**, which exhibited excimers without lipids. We wished to compare these to spectral changes for **R** (isomeric with **C** and **D**) and **I** (structurally similar to **D**). To scrutinize changes most directly, we examined difference spectra: fluorescence emission intensities collected in the absence of SUVs were subtracted from fluorescence emission intensities collected in the presence of SUVs (Figure 4). Although spectra were collected at pH 7.4 and pH 5.0, we noted that intensities changes (measured by *I*_lipid_/*I*_buffer_ values, Table 2) spanned a broader range at pH 5.0. As such, we focused our discussion here on our observations at this pH only. Difference spectra at pH 7.4 for all peptoids and for **M**, **N**, **O**, and **S** at pH 5.0 can be found in Supporting Information Figure S3, accompanied by a description of how pH impacts fluorescence emission.

The effects of SUV addition on the spectra of **B** and **D** were significant. Upon addition of SUVs to **B** and **D** at pH 5.0, the fluorescence emission intensity of the monomer peak increased (positive difference), whereas the intensity of the excimer peak decreased (negative difference) (Figure 4). For **B** and **D**, *I*_lipid_/*I*_buffer_ = 1.39 and 1.71 at 341 nm and 339 nm, respectively, whereas *I*_lipid_/*I*_buffer_ = 0.71 and *I*_lipid_/*I*_buffer_ = 0.45 at 387 nm and 389 nm, respectively. At the monomer *λ*_max_, the fluorescence enhancements were consistent with adsorption of aqueous‐phase peptoids to SUVs. In analogous studies, increased fluorescence emission intensities were observed for coumarin,^62^ quinine,^46^ and tryptophan‐containing peptides^47^ in the presence of SUVs. However, the decreasing intensity of the excimer peak suggested that introduction of SUVs induced a conformational rearrangement in **B** and **D** that disfavored excimer formation.^63^, ^65^ Other studies have established that flexible bi‐naphthalene molecules can exhibit excimers in lipid vesicles.^66^ However, in our case, this conformation was not favored.

Although **A**, **C**, **I**, and **R** did not exhibit excimers, their fluorescence emission intensities were affected differently by the introduction of SUVs. Intensities for the spectra of **A**, **C**, and **R** increased upon addition of SUVs (*I*_lipid_/*I*_buffer_ ratio = 2.68, 2.34, and 2.13, respectively). In contrast, the intensity of the fluorescence emission of the structurally similar but less conformationally mobile tripeptoid **I** exhibited no change (*I*_buffer_/*I*_lipid_ = 0.97 at pH 5.0) when SUVs were added. Increases in fluorescence intensities in the presence of SUVs may be attributed to decreased collisional quenching; chromophores diffuse more slowly through a membrane environment compared to the aqueous phase.^56^ In parallel, we speculated that interaction with SUVs influenced conformation. The conformationally flexible **A**, **C**, and **R** adopted a more rigid structure in the presence of lipids. This in turn reduced non‐radiative relaxation pathways and contributed to higher fluorescence quantum efficiency.^56^ By comparison, the less conformationally mobile **I** did not change structure when SUVs were added to the solution.

To our surprise, the overall charge on the tripeptoid did not correlate with trends in *I*_lipid_/*I*_buffer_. For example, peptoids **A** and **C** had different net charges (+1 and + 2, respectively) due to their different polar side chains but exhibited similarly high *I*_lipid_/*I*_buffer_ values. In previous studies, trends in binding constants likewise did not scale with charge for small molecules adsorbed to DOPC.^67^ Consistent with other studies,^68^ we hypothesized that entropic forces, rather than electrostatic interactions, dominated interaction between small molecules and zwitterionic lipids.

### 3.6 Fluorescence spectra of longer peptoids **T** and **U**

**Figure 5:**
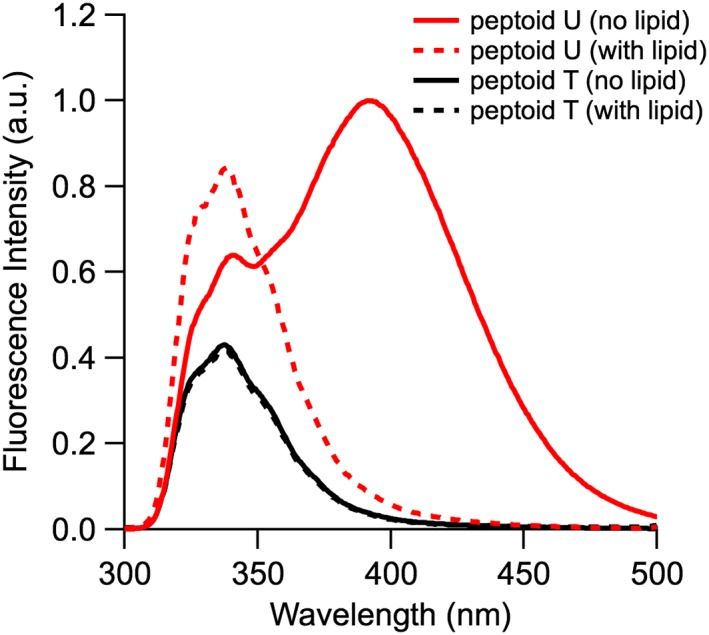
Fluorescence spectra of peptoids **T** (black) and **U** (red) in the absence (solid lines) and presence (dashed lines) of SUVs composed of DOPC. Experiments were conducted at pH 7.4. Dissolved peptoid concentrations were maintained at 40 μM and a lipid‐to‐peptoid molar ratio of 100:1 was used

To explore the effect of peptoid length on spectral changes in the presence of SUVs, the fluorescence emission spectra of 6‐ and 15‐residue peptoids (**T** and **U**, respectively) were also monitored in the absence and presence of 100‐fold excess SUVs composed of DOPC (Figure 5). In the absence of lipid, the emission spectrum of **T** included a single peak and was similar to that observed for the majority of the tripeptoids. The spectrum of **U** in the absence of lipids also included the excimer peak observed for tripeptoids **B** and **D**.

In the presence of SUVs, the fluorescence spectra of **T** and **U** resembled their fluorescence spectra in the organic solvent methanol.^15^ At pH 7.4 in the presence of SUVs, the intensity of the single emission peak of **T** at 340 nm remained unchanged (within error). We expected that **T** would have a single major conformation owing to its two (*S*)‐1‐naphthylethyl side chains and two additional *N*‐α‐chiral substituents that make amide bond isomerization unfavorable.^57^ Similarly, the fluorescence emission of the less conformationally flexible **I** showed little change in the presence of SUVs at pH 5.0 (*I*_lipid_/*I*_buffer_ ≈ 1). Again, the difference in net charge on the peptoids (+1 for **T**, +2 for **I**) did not correlate with spectral changes effected by lipid addition. This observation again highlighted that conformational flexibility was a likely contributor to spectral changes.

Of the peptoids studied here, **U** was the longest and most hydrophobic, and we expected that it would have greatest affinity for lipids. The spectrum of **U** changed dramatically upon addition of SUVs: the 340 nm emission peak increased although the excimer peak disappeared. *I*_lipid_/*I*_buffer_ ratios for the monomer vs excimer peaks were 1.30 and 0.04, respectively (Table 3). Because the excimer emission was previously correlated with self‐association of **U**,^15^ we theorized that the addition of SUVs to **U** decreased its self‐association, either in solution or upon binding to model PMs. Longer peptoids with bulkier, more hydrophobic substituents were previously shown to penetrate bacterial membranes more readily^5^ and adsorb to large unilamellar vesicles (LUVs) at higher surface concentrations compared to shorter sequences.^26^ Likewise, peptoid length appeared to significantly impact peptoid‐lipid interactions here, warranting further investigations in future studies.

**Table 3:** Summary of fluorescence emission observations for longer peptoids

Peptoid	*λ*_max_ a (nm)	*I*_lipid_/*I*_buffer_ b (pH 7.4)
**T**	338	0.97
**U**	340	1.30
	**392**	**0.04**

a *λ*_max_ emission in pH 7.4 buffer in the absence of lipid vesicles.

b Ratio of fluorescence emission intensity at *λ*_max_ for solutions of peptoid in the presence and absence of 100‐fold excess of small unilamellar vesicles. Data for excimer peak are given in bold.

### 3.7 Correlations between peptoid sequence and excimer emission

Correlations between peptoid sequence and conformation both in the absence and presence of lipids will advance the design of peptoids that use naphthalene excimers as structural probes or sensors in biological environments. In the absence of SUVs, we recognized that the emission spectral features are most influenced by the relative positioning of the naphthalenes (i.e., whether or not they are on adjacent residues) and the relative flexibility of the backbone. By contrast, the charge or other properties of the polar side chains did not have a significant impact on emission spectral features. In the presence of SUVs, we also noted that conformational flexibility impacted spectral differences dramatically, whereas charge did not correlate well with intensity changes. From an educational standpoint, we recognized that the richness of the data offered an opportunity to challenge student researchers to consider a range of factors that contributed to our analysis. In doing so, they learned more about peptoid conformation and detailed effects of side chain identity on overall peptoid charge, for example.

### 3.8 Educational outcomes and future iterations of the CURE

Student comments in course evaluations following their participation in the CURE offered insights into the educational outcomes of this work. Overall, student feedback was extremely positive about the research‐focused course. The students' impressions of the CURE were summarized by these representative student comments: “really enjoyed this lab and got a good idea of what research is all about,” “challenged me to learn to learn, not just for a grade,” and “very engaging to work with new kinds of challenging experiments where things don't always go according to plan.” Student feedback indicated that they valued learning new technical skills as part of these research projects. This student feedback was consistent with the learning outcomes reported in other CUREs.^34^

In this project, CURE students' activities were limited to tripeptoid synthesis, but we intend to evolve the laboratory activities in future iterations of this CURE. We will share data obtained here with students and develop exercises that will charge them with proposing new peptoid sequences for preparation. Their participation in these new exercises will give them a richer context for their experimental work. Moreover, CURE students will experience a fuller complement of research activities that more closely parallels those of students in faculty‐led independent research. Although both undergraduate laboratory research assistants and CURE students contributed to reading drafts of this manuscript during its preparation, our experience has been that much of the detailed data analysis and writing still remains the task of faculty.

## 4 CONCLUSIONS

The comparative spectral data discussed here constitute an initial evaluation of peptoid sequence features that influence their photophysical properties in the presence and absence of lipids. We have correlated peptoid sequence features to molecular conformation and evaluated the impacts of SUV introduction on spectral features. In the absence of lipids, peptoid sequence influences spectral features, most substantially by conferring access to a conformation or aggregate that enables excimer fluorescence. In the presence of lipids, we observe changes in fluorescence intensities. Changes suggest that tripeptoids interact with SUVs, and we highlight our observation that overall peptoid charge does not correlate with the magnitude of the changes observed. Importantly, we note major fluorescence spectral changes in excimer‐forming peptoids upon introduction of lipids, consistent with conformational changes in these molecules. These findings will inform the design and implementation of peptoids in new applications that require performance in a complex biological environment. Insights from this work will be useful to advance peptoid therapeutics, coatings, or biosensors where they might encounter cell membranes.

The initial set of results motivates ongoing studies in our laboratories that will provide a more detailed, quantitative picture of the relationships between peptoid structure and their interactions with lipids than the one provided by steady‐state fluorescence spectroscopy. We intend to evaluate peptoid interactions with immobilized model PMs using second harmonic generation (SHG) spectroscopy. Use of SHG spectroscopy will allow us to detect low (and biologically relevant) concentrations of peptoids adsorbed to diverse immobilized lipids and to quantify the thermodynamic forces which drive peptoid‐lipid interactions.

Through this work, we also demonstrate that undergraduate researchers can be important contributors to advancing peptoid science. Even without much experience as experimentalists, undergraduates can reliably execute approachable techniques, including solid‐phase synthesis and fluorescence spectroscopy. We prioritize developing exercises or creating venues that cultivate students' understanding of the broader context for their individual contributions to the project when engaging students in research activities. In our experience, students are motivated by working to achieve a “big picture” goal that addresses an important challenge that they recognize. We advocate for the incorporation of peptoid synthesis experiments into instructional laboratories for investigators who need access to large numbers of diversely functionalized peptoids. Enrolled students are an under‐utilized “workforce,” and the synergistic research and educational benefits from engaging students in research modules are compelling. Students trained in these research‐based courses are also excellent candidates to continue in faculty‐led research, including at the graduate level.

## AUTHOR CONTRIBUTIONS

Tan, Dowell, Burgess, Cervantes, Chan, Jandaur, Karanik, Lee, Ley, McGeehan, McMonigal, Palazzo, Parker, Payman, Soria, Verheyden, Vo, and Calkins prepared and purified peptoids described here in the CURE laboratory course and/or in faculty‐led research. Jimenez, Gadbois, Read, Calkins, and Yin prepared and characterized SUVs and collected fluorescence spectra on peptoids. Jimenez, Gadbois, Read, Calkins, Fuller, and Stokes analyzed the data. Fuller and Stokes wrote the manuscript with feedback from all authors.

## Supporting information

Appendix S1: Supporting information
