# Supplementary material for: Peptoids advance multidisciplinary research and undergraduate education in parallel: Sequence effects on conformation and lipid interactions
Source: Biopolymers. 2019 Jan 11;110(4):e23256. doi: 10.1002/bip.23256 (PMC6590334; doi:10.1002/bip.23256)
Supplement: Supplementary file 1 — Appendix S1: Supporting information [file BIP-110-na-s001.pdf]

Supporting information for:

Peptoids advance multidisciplinary research and undergraduate education in parallel: Sequence effects on conformation and lipid interactions

Christian J. Jimenez, Jiacheng Tan, Kalli M. Dowell, Gillian E. Gadbois, Cameron A. Read, Nicole Burgess, Jesus E. Cervantes, Shannon Chan, Anmol Jandaur, Tara Karanik, Jaenic J. Lee, Mikaela C. Ley, Molly McGeehan, Ann McMonigal, Kira L. Palazzo, Samantha A. Parker, Andre Payman, Maritza Soria, Lauren Verheyden, Vivian T. Vo, Jennifer Yin, Anna L. Calkins, Amelia A. Fuller,* Grace Y. Stokes*

Department of Chemistry & Biochemistry, Santa Clara University, Santa Clara, CA 95053, USA

Co-corresponding Authors:
*Email; aafuller@scu.edu and gstokes@scu.edu

Table of contents:

Item	Page number
**Table S1.** High resolution mass spectral data for representative tripeptoids	S2
**Table S2.** Extinction coefficients for peptoids	S2
**Figure S1.** Comparison of fluorescence of **D** at two different solution phase peptoid concentrations	S3
**Figure S2.** Fluorescence emission spectra of tripeptoids with lipids	S4
pH impacts on fluorescence spectra in the presence of lipids	S5
**Figure S3.** Difference fluorescence emission spectra for **M**, **N**, **O** and **S** at pH 7.4 and pH 5.0	S5
**Figure S4.** Difference fluorescence emission spectra for **A**, **B**, **C**, **D**, **I** and **R** at pH 7.4	S6
Student instructions for synthesis of tripeptoids experiment	S7-S18
Background	S7-S8
Your goal	S8
Preparation	S9
Procedures for peptoid synthesis	S10-S13
Introduction to LC/MS	S14-S15
Looking at LC/MS data	S16-S17
Instructions for peptoid presentations	S18
Instructor materials for synthesis of tripeptoids experiment	S19-S20
Chemical Information and Hazards	S19
General Hazards	S20
Equipment and Consumables	S20
Instrumentation	S20
Preparation	S20-S21
Specific Notes	S21
LC/MS conditions	S21

Table S1. High resolution mass spectral data for representative tripeptoids

Peptoid	Calculated m/z for $[M+H]^+$	Observed m/z
**A**	527.2658	527.2656
**D**	512.2662	512.2660
**I**	568.3288	568.3285
**M**	506.2767	506.2784
**N**	513.2502	513.2499
**O**	571.2920	571.2915
**R**	512.2662	512.2656
**S**	527.2294	527.2291

Table S2. Extinction coefficients measured for tripeptoids

Peptoid	molar extinction coeffiient (ε) at 266 nm in 5 mM Tris buffer, pH 7.5 ($M^{-1}cm^{-1}$)
**A**	7190
**B**	7028
**C**	4722
**D**	2715
**I**	7243
**M**	2490
**N**	4258
**O**	3953
**R**	4036
**S**	6473

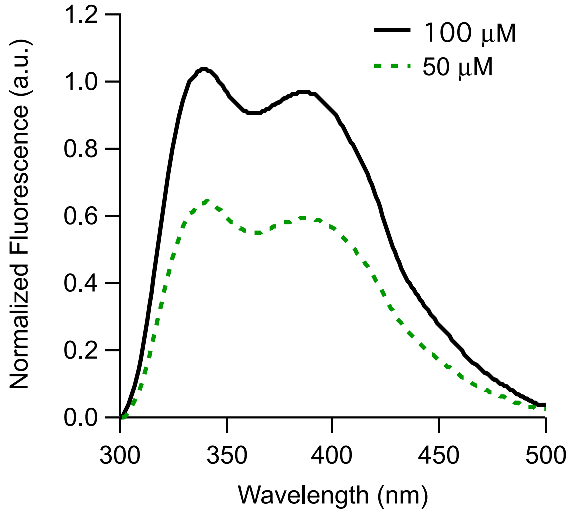

Figure S1. Fluorescence emission spectra of **D** at two different solution phase peptoid concentrations (at pH 7.4). Spectral features do not change over the concentration range.

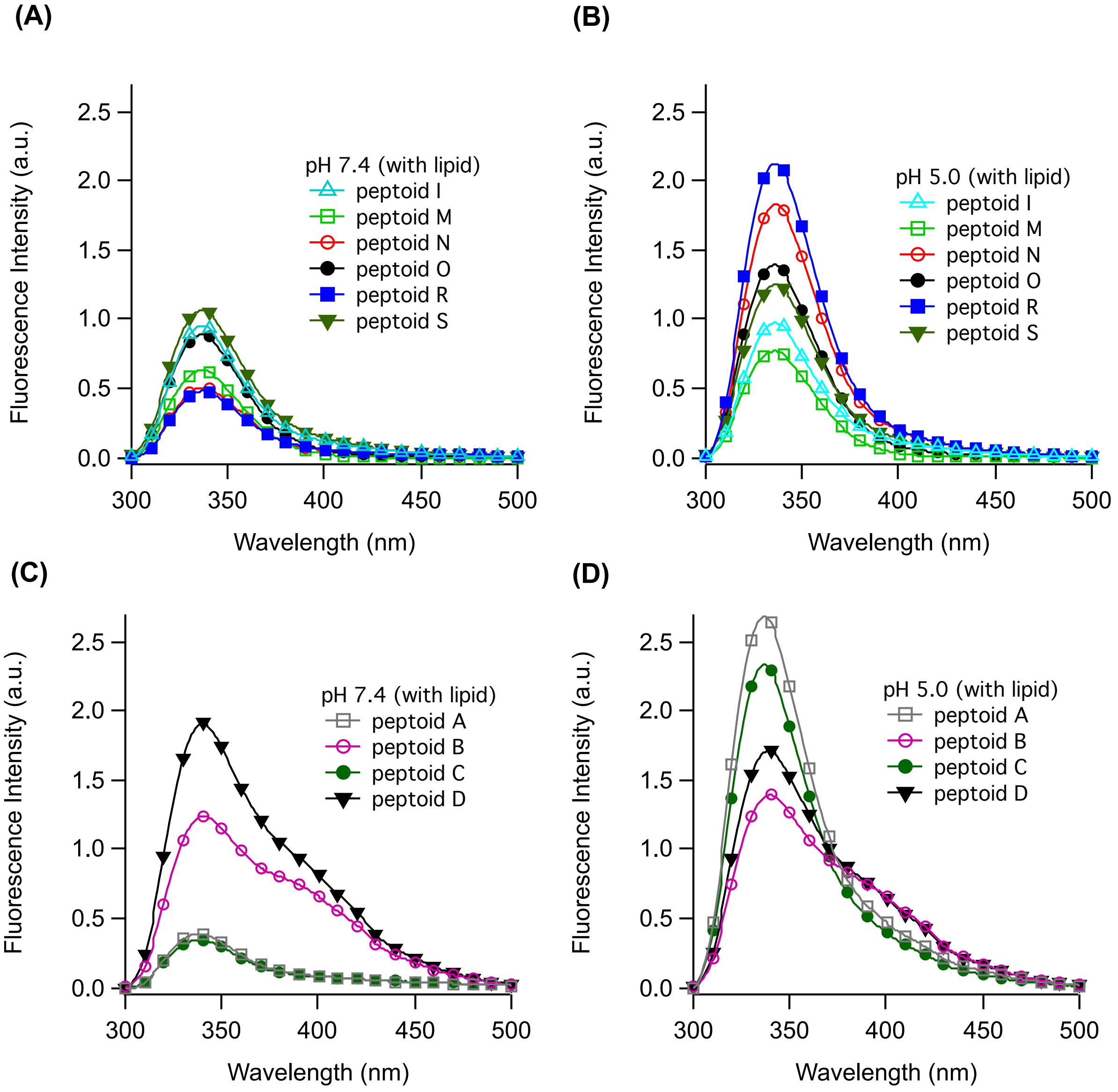

Figure S2. Fluorescence emission spectra of tripeptoids **I**, **M**, **N**, **O**, **R**, **S** (panels A and B) and **A**, **B**, **C**, and **D** (panels C and D) in the presence of small unilamellar vesicles (SUVs) composed of DOPC. Experiments were conducted at pH 7.4 (panels A and C) and at pH 5.0 (panels B and D). Dissolved peptoid concentrations were maintained at 100 μM. A lipid-to-peptoid molar ratio of 16.6 : 1 was used for all peptoid sequences. Data were normalized to the fluorescence emission intensities of each peptoid sequence at pH 5.0 in the absence of SUVs.

pH impacts on fluorescence spectra in the presence of lipids

Fluorescence emission intensities in the presence of lipids were generally lower at pH 7.4 than at pH 5.0, which is evidenced by $I_{5.0}/I_{7.4}$ (with lipid) > 1 values in Table 2 in the main manuscript. Consistent with our observations in the absence of small unilamellar vesicles (SUVs), we observed the highest $I_{5.0}/I_{7.4}$ for **A**, **C**, **R** and **N** in the presence of SUVs. As such, we attributed this trend to higher fluorescence quenching by neutral amines at higher pH. The lowest $I_{5.0}/I_{7.4}$ was observed for **D**, although structurally similar **B** and **I** also exhibited minimal changes in fluorescence intensities as a function of pH in the presence of SUVs. In the case of **D,** $I_{5.0}/I_{7.4}$ = 0.82 for the excimer emission peak and $I_{5.0}/I_{7.4}$ = 0.90 for the lower wavelength emission in the presence of SUVs. Our observations are consistent with previously published studies involving liposomes that showed increased fluorescence emission intensities from bound chromophores when pH was decreased.

References: D. W. Deamer, R. C. Prince, A. R. Crofts, *Biochim. Biophys. Act.* **1972**, *274*, 323.
R. A. Parente, L. Nadasdi, N. K. Subbarao, F. C. Szoka. *Biochemistry* **1990**, *29*, 8713.

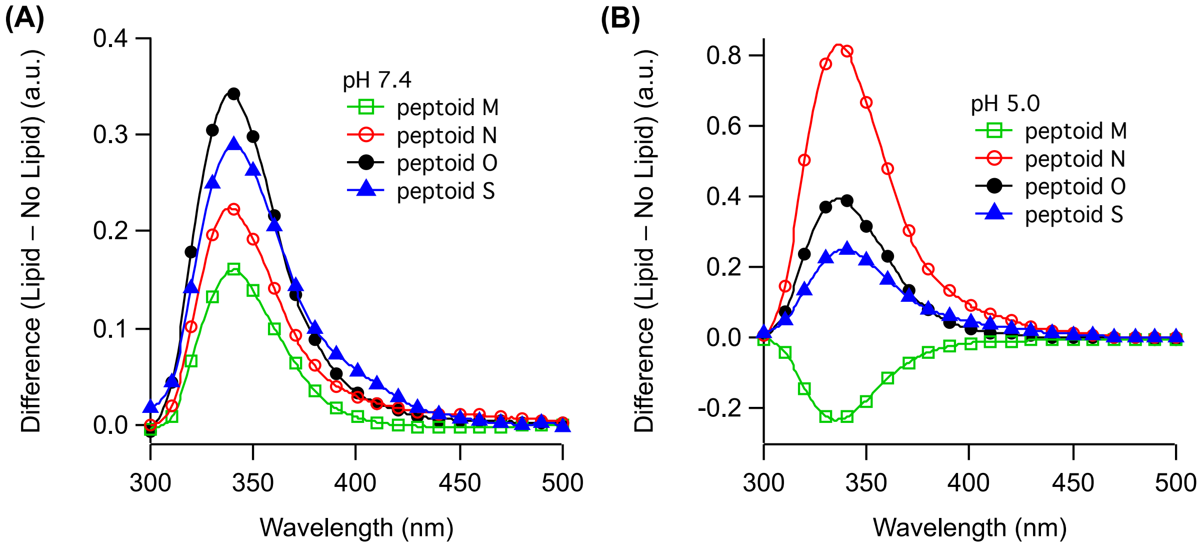

Figure S3. Difference in fluorescence emission intensities in the presence minus in the absence of SUVs composed of DOPC for tripeptoids **M**, **N**, **O** and **S**. Experiments were conducted at pH 7.4 (panel A) and at pH 5.0 (panel B). Dissolved peptoid concentrations were maintained at 100 μM. A lipid-to-peptoid molar ratio of 16.6 : 1 was used for all peptoid sequences.

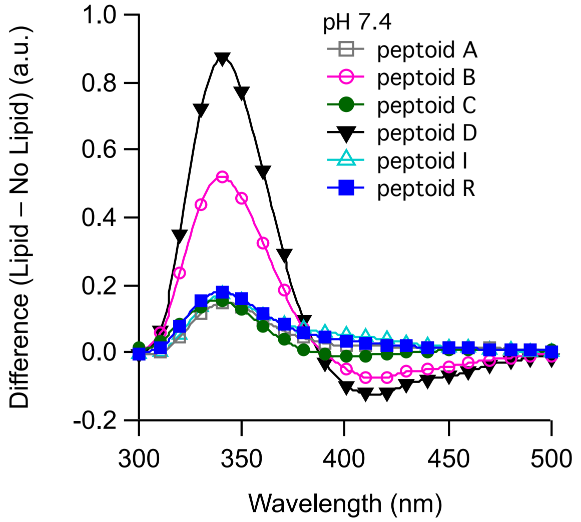

Figure S4. Difference in fluorescence emission intensities in the presence minus in the absence of SUVs composed of DOPC for tripeptoids **A**, **B**, **C**, **D**, **I** and **R**. Experiments were conducted at pH 7.4. Dissolved peptoid concentrations were maintained at 100 μM. A lipid-to-peptoid molar ratio of 16.6 : 1 was used for all peptoid sequences.

BACKGROUND

Peptoids. Peptoids (*N*-substituted glycine oligomers) were originally conceptualized as mimics of peptides (amino acid oligomers). In the side-by-side comparison of peptides and peptoids, the variable "R" groups of the peptide side chains have been relocated to the amide nitrogen in the peptoid. This structural change modifies many of the properties of peptides. For example, peptoids do not have hydrogen bond donors as peptides do; as such, their structures are different from those of peptides. Additionally, if used in a biological context, peptoids are not metabolized in the same way as peptides. Although peptides are quickly digested by proteins in cells, peptoids stick around longer. This is very useful if you want the peptoid to interact with a biological target (e.g, a protein).

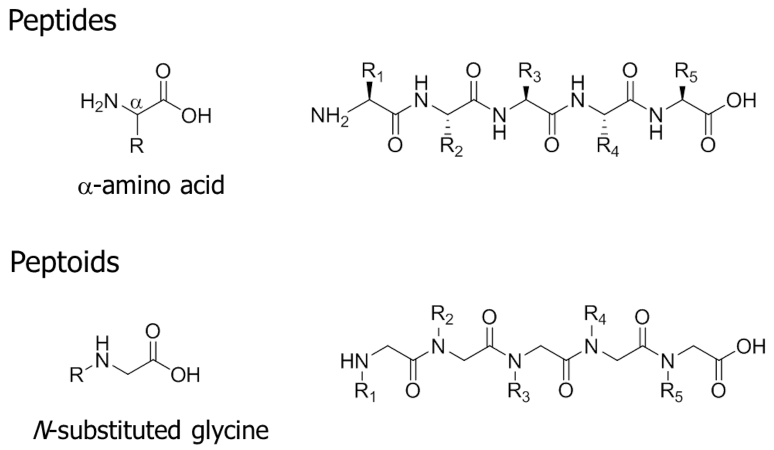

Perhaps more substantially, the ease of synthesis of peptoids has attracted many researchers to try to use them in a variety of applications, some of which you'll hear more about in this class. The synthesis method is called the "submonomer synthesis." Each *N*-substituted glycine unit of the molecule is built in two high-yielding reaction steps. Additionally, each unit is installed step-wise, which allows the synthetic chemist to control the sequence in which these building blocks are added. The variable parts of the peptoids (the R groups) come from primary amines. If you take a look at a chemical catalog like the Aldrich catalog, there are many, many amines that you can choose to buy, and most will work well in these reactions.

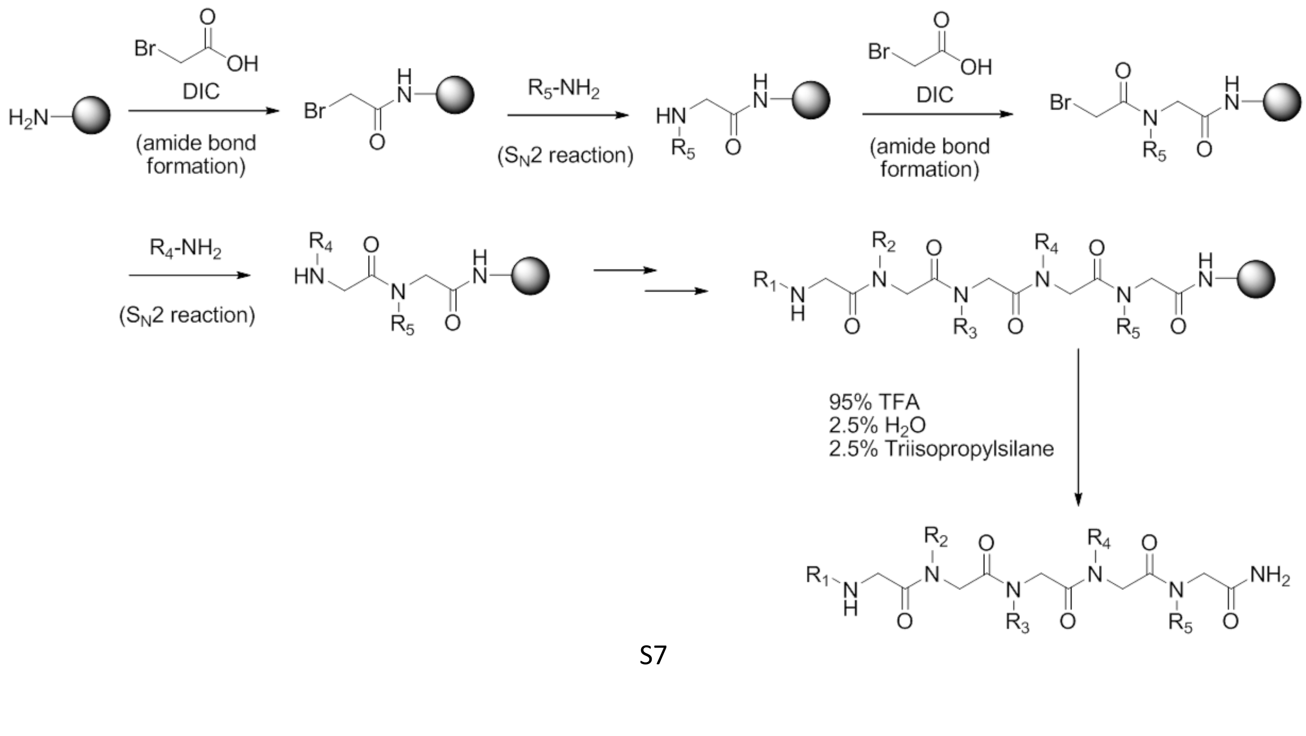

Combinatorial Chemistry. Combinatorial chemistry is an age-old approach to problem solving. It is most powerfully exemplified in nature where a simple set of 20 amino acids, biochemically arranged in a combinatorial fashion, affords the millions of peptides and protein structures required for life. Organic and peptide chemists are now applying these same approaches in the laboratory. According to Curran and Wipf (Chemical & Engineering News, p. 7, March 17, 1997) “Combinatorial synthesis is the intentional construction of a collection of molecules based on logical design and involving the selective combination of building blocks by means of simultaneous chemical reactions. The collection of molecules resulting from a combinatorial synthesis is a combinatorial library.”

Solid-Phase Chemistry. In combinatorial chemistry the need to carry out these multiple reactions on a small scale made the use of solid-phase chemistry attractive, and there has been much recent work on adapting traditional organic chemistry to the solid phase. Solid-phase chemistry was pioneered by Professor Bruce Merrifield in 1963. For this work he received the Nobel Prize in Chemistry in 1984. He showed that the small scale (10-100 μmol) multistep synthesis of polypeptides could be simply and effectively carried out on a solid support, polystyrene beads, functionalized with a specific reactive group. The reactive group attached to the beads will undergo chemical modification. Because the insoluble beads don’t pass through a glass frit, the easy filtration work-up at each step permits the use of excess reagents, difficult solvents and, eventually, automation of the complete synthetic process. At the end of the synthesis, the product is cleaved from the polystyrene beads. The beads are then separated from the desired product by filtration.

Because you often don’t isolate intermediate reaction products along the way, you can’t check the Rf by TLC or run a GC on your intermediate products like you might if you were doing solution phase chemistry. Instead, chemists often use colorimetric tests to check for the presence or absence of a particular functional group in their product. You’ll use this strategy here to check for the presence or absence of secondary amines, as shown in the scheme below. A secondary amine reacts with acetaldehyde and chloranil to form a blue-green colored product.

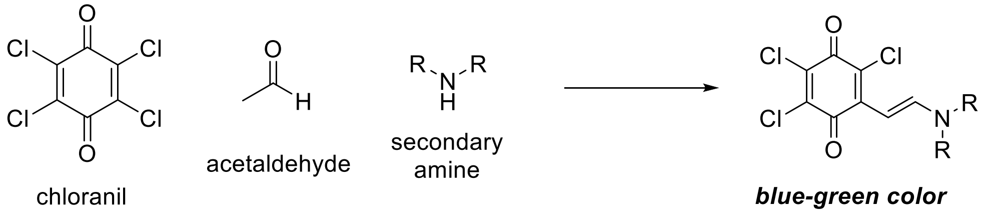

YOUR GOAL

In this experiment, your goal is to make a sequence-specific three-residue peptoid for further study in a research lab in the department. You’ll be assigned a specific structure to make (I will define the “R” groups). We’ll talk more about how the peptoid structures you’ve been assigned were chosen.

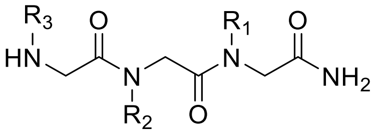

PREPARATION FOR PEPTOID SYNTHESIS EXPERIMENTS

Know the hazards associated with your procedures:

All of the chemicals we use in this lab are hazardous. As usual, wear protective equipment and work in the fume hoods at all times. Products that we make are unknown molecules and may have biological activity. Every care should be taken to minimize your exposure to these compounds.

Read through the procedures carefully before you begin work. Specific chemical hazards associated with the relevant steps are identified in the procedure.

Calculations worksheet:

You'll have to prepare a solution of at least one of your amines for use in the synthesis (step 5 in the procedures below). *We'll have a solution of 1-napthylmethylamine already made up*.

Amine solutions should be at a concentration of 1 M in *N*-methyl-2-pyrrolidone (NMP) solvent. You'll need 2 mL of solution for your reaction, although you can make a bit extra if it makes measurements easier. To help you get ready to make your solution(s), answer the questions on this worksheet and turn it in to your instructor by *[insert due date and submission instructions]*.

> Tips: Looking up molecules' names and properties on the bottle labels or on Aldrich.com will be helpful here. Also, many of your side chain contains an amine or an alcohol; these functional groups will be protected until the final synthesis step (cleavage from the resin). Amines will be protected with the "BOC" group, and alcohols will be protected with the "TIPS" group. See me if you need help finding these structures.

1. What peptoid are you making (give the letter code)?
2. How many amines solutions do you need to make?
3. For each amine, answer the following:

What is the name of the amine you'll use?
What is its structure? (draw in by hand or with chemdraw)
Is it a liquid or a solid?
What is its molecular weight?
What is its density (if available)?
To make 2 mL of a 1 M solution, how many moles of amine do you need?
How many mg (for solid) or mL (for liquids) of your amine do you need for your solution?
How much NMP will you add to have a total solution volume of 2 mL?

SYNTHESIS PROCEDURES

General washing procedure: draw 2 mL of the specified solvent into the syringe. Shake once, then expel the solvent from the syringe into your waste beaker.

1. Swell the resin

Hazards: Rink amide resin is an irritant, DMF is flammable, is an irritant, and is a health hazard.

a. You will receive a syringe pre-loaded with 0.1 mmol Fmoc-Rink amide resin.
b. Set up two beakers: one with approximately 50 mL N,N-dimethylformamide (DMF) and one labeled "waste."
c. Draw 2 mL DMF into the syringe. Expel it out into the waste beaker. Repeat 2 more times.
d. Draw 2 mL DMF into the syringe and cap it.
e. Allow the resin to sit undisturbed for at least 10 minutes (we'll do something else while the resin swells). This swells the resin, opening up its porous center so its reactive functional groups are exposed and available to the reagents we'll add.

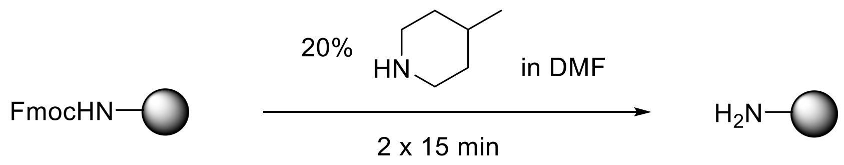

Fmoc-Rink amide resin

2. Deprotect the amine (remove Fmoc)

Hazards: DMF is flammable, is an irritant, and is a health hazard. 4-methylpiperidine is flammable and an irritant.

a. Uncap, then expel the DMF from the syringe
b. Draw 2 mL of the prepared 20% 4-methylpiperidine in DMF solution into your syringe.
c. Cap the syringe, and shake it gently.
d. Allow the syringe to sit for at least 15 minutes.
e. Uncap, then expel the solution from the syringe into the waste.
f. Wash with DMF two times.
g. Repeat steps b-e
h. Wash with DMF 10 times. Expel the DMF from the syringe.

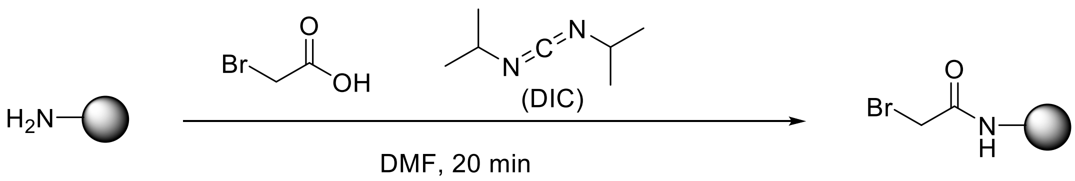

3. Bromoacetylation

Hazards: DMF is flammable, is an irritant, and is a health hazard. Bromoacetic acid is corrosive, toxic, and an environmental hazard. DIC is flammable, corrosive, toxic, and a health hazard. NMP is an irritant and health hazard.

a. In a small vial, combine 1.7 mL of the 1.2 M bromoacetic acid solution with 0.4 mL DIC. Make this solution JUST BEFORE you use it.
b. Draw the solution you made in part a into the syringe.
c. Cap the syringe and shake it gently.
d. Shake the syringe occasionally over 20 mins.
e. Uncap, then expel the solution from the syringe into the waste.
f. Wash with DMF 4 times, then with NMP 4 times.

4. Monitor reaction completion: chloranil test

Hazards: Rink amide resin is an irritant. DMF is flammable, is an irritant, and is a health hazard. Chloranil is an irritant and environmental hazard. Acetaldehyde is flammable.

a. Carefully remove the plunger from the syringe.
b. Using a glass pipette, remove just a few resin beads, and transfer them to a test tube.
c. Replace the plunger on the syringe.
d. To the **test tube**, add a few drops (approx. 5) of each chloranil reagent solution, A and B.
e. Wait 5 min, then inspect the color of the **beads** (not the solution). Blue/green beads indicate that a secondary amine is present. Colorless beads indicate no secondary amine. **Which do you expect here to verify that your reaction has worked?**

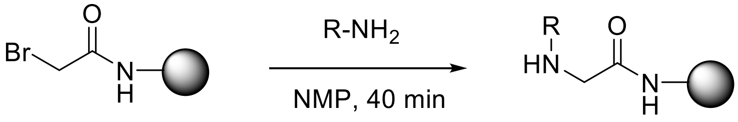

5. Amine displacement

Hazards: NMP is an irritant and health hazard. Most amines are corrosive and several are flammable. DMF is flammable, is an irritant, and is a health hazard.

a. Draw 2 mL of the 1 M solution of the appropriate amine into the syringe.
b. Cap the syringe and shake it gently.
c. Shake the syringe occasionally over 40 mins.

d. Uncap, then expel the solution from the syringe into the waste.
e. Wash with DMF 8 times.

6. Monitor reaction completion: chloranil test

Hazards: Rink amide resin is an irritant. DMF is flammable, is an irritant, and is a health hazard. Chloranil is an irritant and environmental hazard. Acetaldehyde is flammable.

a. Repeat steps listed under part 4. **What color do you expect the beads to be to verify that your reaction has worked?**

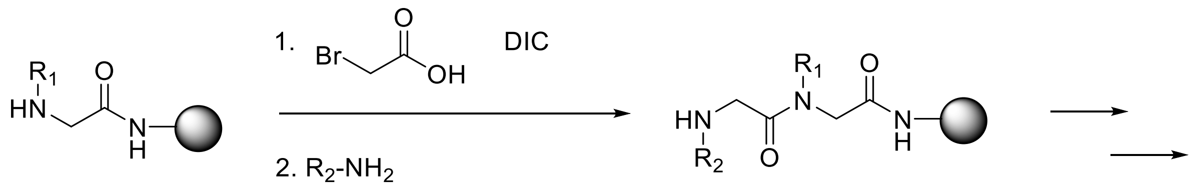

7. Iterate the synthesis to extend the peptoid chain length.
   a. Repeat steps 3-6 using the appropriate amine solutions in step 5 for each iteration.

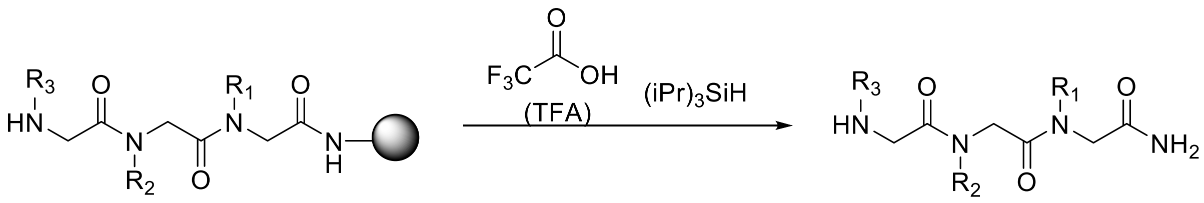

8. Cleavage from the resin and deprotection of side chain functionality.

Hazards: Dichloromethane is an irritant and health hazard. ***TFA is a VERY STRONG ACID****. It is corrosive and an irritant. Triisopropylsilane is flammable.*

a. Wash the resin 5 times with CH_2Cl_2
b. Carefully remove the plunger from the syringe.
c. Let the resin dry in the air for 10 mins.
d. Cap the syringe and clamp it in your hood with the top open.
e. Carefully add the TFA mixture provided to the resin.
f. Let this mixture sit for 60-90 mins.
g. Meanwhile, label a 20-mL glass vial with your initials and your peptoid's structure.
h. Find the tare weight of the empty vial and record it in your notebook.
i. After 60-90 mins, quickly uncap the syringe, and collect the liquid in the tared, labeled vial.

9. Prepare a sample for LC/MS analysis

Hazards: methanol is flammable and toxic.

a. Take1- 2 drops of the solution of your crude mixture and place it in an LC/MS vial labelled with your initials and your peptoid's molecular weight.
b. Fill the vial with methanol.
c. Give the vial to professor to run the LC/MS experiment.

Introduction to LC/MS

Liquid Chromatography/Mass Spectrometry (LC/MS) techniques allow us to get information on the relative purity and the identity of components in a mixture. The LC is responsible for separating mixture components. They move in solution through a column containing a stationary phase at different rates, then onto two different detectors that enable us to monitor this separation. A UV detector measures the absorbance of each component at a given wavelength. The MS detector provides a mass spectrum of each of the mixture components, enabling us to identify which component that eluted has the desired molecular mass.

LC = liquid chromatography

Separates molecules based on their affinity for the stationary phase vs. the mobile phase
Generally correlates with polarity of molecules

Normal phase:
Stationary phase is polar; mobile phase (eluent) is less polar
Examples: TLC plates, silica gel columns
Stationary phase is silica (SiO_2)
Common mobile phases includes hexane, ethyl acetate, CH_2Cl_2
More polar molecules have higher affinity for stationary phase. This translates to lower R_f's on TLC plates, or later elution on silica gel columns for more polar molecules.

Reverse phase:
Stationary phase is nonpolar; mobile phase (eluent) is polar
Example: column on LC/MS instrument
Stationary phase is C18 (silica functionalized with long hydrocarbon chains)
Common mobile phases include water, methanol, acetonitrile (CH_3CN)
More polar molecules have lower affinity for the stationary phase, so they move through the C18 column more quickly. This translates to lower retention times (t_R) for more polar molecules.

UV detector

On our instrument, after being separated by the C18 column, molecules are passed through a UV detector set to detect absorbance at 254 nm. Any molecule with an aromatic ring will have some absorbance at 254 nm (note that your molecules definitely have at least two aromatic rings). This detection method can be done on the molecules in solution and does not destroy or damage the compounds. So, the solution can pass through the UV detector and onto the second MS detector.

MS detector

The second detector is an MS detector, which will generate a mass spectrum for each of the components that elutes from the LC column. Recall that mass spectrometry detects charged species (ions). Specifically, we'll be detecting cations. There are a number of ways to make ions, one of which (electron impact) we introduced briefly in lecture. The MS detector on our instrument generates ions directly from the eluting liquid solution by electrospray ionization.

Electrospray Ionization

Stage 1

The sample is mixed with a solvent and introduced into a vessel, called a capillary, that ends in a very fine tip. A very high voltage is applied to this tip, which charges the molecules in the solvent. Because they are highly charged with the same charge, these molecules, once they are pushed through the nozzle into the evaporation chamber, repel one another almost violently. When the charged liquid first exits the tip, it briefly forms a cone shape (known as a Taylor cone) before the droplets burst away from each other into a fine spray.

Stage 2

Released from the nozzle, the droplets in the spray go through a series of divisions. This occurs because the solvent within these droplets gradually evaporates (with the assistance of nitrogen gas pumped into the chamber), forcing the charges in the molecules within these droplets (which, again, are identical) closer together. When these ions are pushed close enough together, they will repel each other (this behavior is known as the Coulomb force), causing the droplets to divide into two smaller droplets. This process repeats itself until the solvent is completely evaporated and the droplets have split up to the point that each is a single, charged molecule. One of the advantages of this ionization method is that the molecules remain intact and will not be broken apart; this is what is meant when this type of ionization is called “soft.”

Looking at your LCMS data:

Here's an example of the chromatograms you'll receive and what information you get from them:

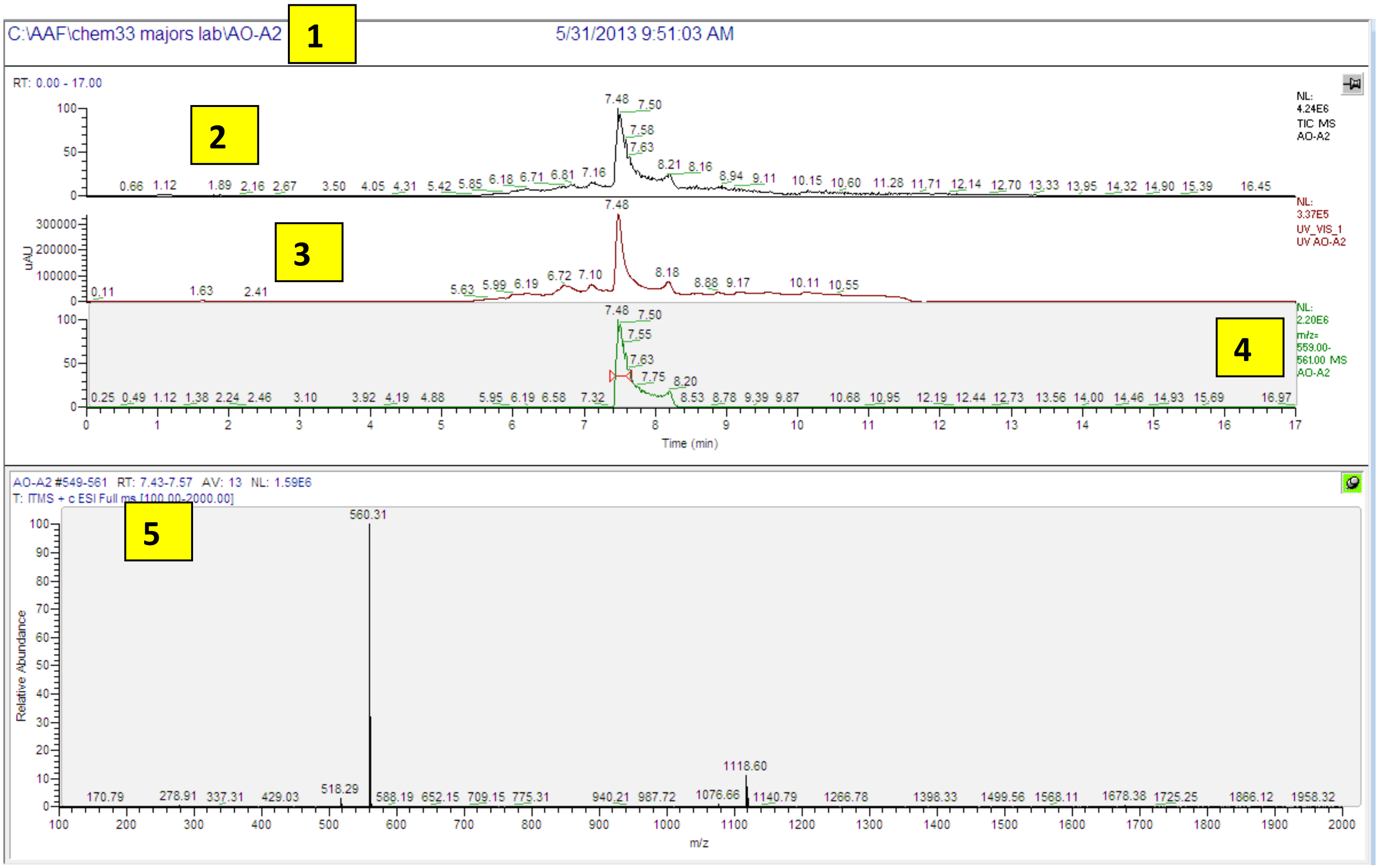

1. Sample ID—this should match the information you provided.
2. Total ion chromatogram (TIC): MS signal plotted vs. time. This is where the MS detector sees ANY mass/charge signal 100-2000 AMU.
3. UV chromatogram: Signal vs. time plot where the UV detector sees any species that absorbs light at 254 nm, indicating that the molecule that generates that peak has an aromatic ring.
4. Extracted ion chromatogram. Anywhere in the time plot where the MS detector sees a certain mass/charge range. This m/z range on the right side of the plot in very small print (here it's 559-561). It should correlate to the mass of your compound from the chemdraw files you generated (plus 1 for the mass of the H+).
5. Mass spectrum at a certain time or time range. The time range is at the top of the mass spectrum and also shown by the horizontal red bar in one of the chromatograms above (here it's 7.43-7.57 min). If you have multiple data shown for a sample, I've given you the mass spectrum signal at more than one peak in the chromatogram, so this retention time range will change (as will the placement of the red bar in the chromatograms). I can get you more data if you want to see other peaks—just ask.

Things to consider when analyzing your LCMS data:

Address all of these in your notebook as you write up the results of your experiments.

1. Did your reaction work? Did you make your desired products?
2. Did your reactions work well? Is your desired product one of the main components of the crude mixture?
3. If you have multiple components in the mixture, can you identify any of the byproducts based on their masses plus any other information you have from your procedures? What other structures did you consider?
4. Do you have any suggestions for changes to the procedures or reagents used that might improve the outcomes of the reactions?

Instructions for peptoid presentations

Your group will give two presentations pertaining to peptoids. These are meant to be informal and developmental--they are intended to get you used to talking about the experiments you're working on and get feedback from me and your peers about how to present things clearly. Presentations are *not* oral exams. If you want me to check over anything before your presentation, I'm happy to do it as long as I get it by 9am Tuesday the day before your presentation.

1. <u>Peptoid application presentation</u>

Your group will make a brief presentation (5-10 min) and accompanying slide(s) to tell the group about how peptoids have been used by other scientists. Through this exercise, you will learn:

- some of the uses of peptoids in diverse chemistry research
- extracting critical information from papers
- making a brief presentation, including the use of chemdraw software

Instructions:
Read the paper assigned to your group, then prepare a five-minute presentation in which you address the following:

- Briefly summarize the goal of the work. (only a few sentences—nothing deeply detailed)
  For example: The Smith lab was trying to find a peptoid that treats toenail fungus infections. They made and tested peptoids that mimic antifungal peptides.
- Which 1-3 peptoid(s) was/were found to work best in the experiments described?
- Draw the structure(s) in chemdraw, and show them in your presentation.
- What structural features of the peptoid were important for its function in the experiments (if known)?

Save your presentation slide(s) in the shared Google Drive folder.

I know it's hard not to get bogged down in the many, many details of a research paper. I expect that you won't understand many of the techniques (I don't understand them all!)--that's not the point of this assignment. Just try your best to distill out the most important result and the peptoid structure(s) at the heart of this. See me with your questions.

2. <u>Mechanisms of peptoid synthesis</u>

Your group will make a brief presentation (5 min) and accompanying slide(s) to tell the group about the mechanism of reactions used in the synthesis of peptoids. Through this exercise, you will learn:

- Making a brief presentation, including the use of ChemDraw software
- Searching for new reaction mechanism information
- Mechanism details for reactions that we run in the lab; this is important knowledge to know how to troubleshoot when reactions don't work well.

Instructions: Using ChemDraw, illustrate the step-wise mechanism using curved arrows to represent electron movement. In your presentation, talk through each step to explain it to the class. Post your slide(s) in the shared Google drive folder.

Chemical Information and Hazards:

Chemical	CAS number	Quantity per student	Hazards
Rink amide resin, 100-200 mesh, copoly (styrent-1% DVB), typical loading 0.74 mmol/g (EMD Millipore, catalog # 855001)	[9003-70-7]	Approx. 135 mg§	Irritant
N,N-dimethylformamide (DMF)	[68-12-2]	70 mL	Flammable, irritant, health hazard
4-methylpiperidine	[626-58-4]	5 mL	Flammable, irritant
Bromoacetic acid	[79-08-3]	1 g	Corrosive, toxic, environmental hazard
N,N'-diisopropylcarbodiimide	[693-13-0]	1.4 mL	Flammable, corrosive, toxic, health hazard
Methylpyrrolidone (NMP)	[8725-50-4]	6 mL	Irritant, health hazard
triisopropylsilane	[6485-79-6]	0.25 mL	flammable
Trifluoroacetic acid	[76-05-1]	5 mL	Corrosive, irritant
Acetaldehyde	[8032-32-4]	Trace	Flammable
p-chloranil	[118-75-2]	Trace	Irritant, environmental hazard
2-methoxyethylamine*	[109-85-3]	1 mL	Flammable, corrosive
4-(2-aminoethyl)morpholine*	[2038-03-1]	1 mL	Corrosive, irritant
1-(2-aminoethyl)piperidine*	[27578-60-5]	1 mL	Flammable, corrosive
1-(2-aminoethyl)pyrrolidine*	[7154-73-6]	1 mL	Flammable, corrosive
N,N-dimethylethylenediamine*	[108-00-9]	1 mL	Flammable, corrosive, irritant
butylamine	[109-73-9]	1 mL	Flammable, corrosive, acute toxicity
2-((triisopropylsilyl)oxy)ethan-1-amine [1]	[158198-43-7]	1 mL	Unknown
2-(2-methoxyethoxy)ethan-1-amine [2]	[31576-51-9]	1 mL	Unknown
2-(2-(2-methoxyethoxy) ethoxy)ethan-1-amine [2]	[74654-07-2]	1 mL	Unknown
N-Boc-ethylenediamine	[57260-73-8]	1 mL	Corrosive
N-Boc-1,3-propanediamine	[75175-96-0]	1 mL	Corrosive, irritant
N-Boc-1,4-butanediamine	[68076-36-8]	1 mL	Corrosive
(*S*)-(−)-1-(1-Naphthyl)ethylamine	[10420-89-0]	1 mL	Irritant
1-naphthylmethylamine	[118-31-0]	2 mL	Irritant
Glycine *tert*-butyl ester hydrochloride salt	[27532-96-3]	1 g	None listed
Alanine *tert*-butyl ester hydrochloride salt	[13404-22-3]	1 g	None listed
4-Phenylbenzylamine	[712-76-5]	1 mL	Irritant
4-Methoxybenzylamine	[2393-23-9]	1 mL	Corrosive, irritant
2-Phenoxyethylamine	[1758-46-9]	1 mL	Corrosive
Dichloromethane	[75-09-2]	5 mL	Irritant, health hazard
Methanol	[67-56-1]	1 mL	Flammable, acute toxicity

§ exact amounts vary depending on resin loading, which is variable from one batch to another.

* these calculations assume that every student will use each of these reagents. Actual amounts needed will likely be somewhat lower depending on precise structures to be prepared, but it is best to have extra of these reagents on hand.

[1] prepared according to the procedure reported in: Zuckermann, R. N., *et al*. *J. Med. Chem*. **1994**, *37*, 2678-2685

[2] provided by the laboratory of Ronald Zuckermann as reported in: Sun, J.; Stone, G. M.; Balsara, N. P.; Zuckermann, R. N. *Macromolecules* **2012**, *45*, 5151-5156.

General hazard information:

Because all chemicals used in these experiments are hazardous, students should have prior laboratory experience and safety training. Work should be carried out exclusively in fume hoods with sashes pulled to the lowest possible position to allow work, and students and instructors should wear gloves, lab coats, pants, shoes, and goggles at all times. Proper syringe technique should be demonstrated and used. Many products made in this laboratory are new compounds; they should be assumed to have biological activity, and direct contact should be strictly avoided.

Equipment and consumables:

Item	Quantity per student	Vendor, catalog # (for specialty items only)
2.5-mL disposable syringe reaction vessel with cap	1	Torviq, SF-0250 (torviq.com, accessed Aug 9, 2018)
Autosampler vials and caps for LC/MS samples	1	
20 mL vials for preparation of reaction solutions and collection of crude tripeptoid product	5	
1 mL syringes to measure volumes of amines in solution preparation	Approx. 10	
Glass pipettes and bulbs	20	
Test tubes for chloranil test	10	

Instrumentation:

Access to liquid chromatography-mass spectrometry system (details below).

Preparation:

Instructor or assistant should add 0.1 mmol resin to each reaction syringe before distributing to students.

Synthesis of non-commerically available amines may be carried out according to literature procedures (see citations in chemical information table above).

<u>Solution preparation</u>: *Solutions should be prepared freshly for each laboratory in which they will be used*.

1. Chloranil test solutions to check reaction completion:

   2% acetaldehyde in DMF: 0.6 mL acetaldehyde, 25 mL DMF (store at 4 °C)
   2% chloranil in DMF: 485 mg chloranil, 25 mL DMF. Shield this solution from light, and store at 4 °C for ≤ 3 days).
   This should be sufficient volume of solution for up to 18 students.

2. 1-naphthylmethylamine solution (2 M in NMP)—*optional*

   For each usage of this amine by any student in the class, make 2.2 mL.

We elected to prepare this solution for all students because everyone had this residue in his/her tripeptoid. In other iterations of this or similar experiments, we have charged students with preparation of all amine solutions.

3. 95% TFA/2.5% H_2O/2.5% triisopropylsilane solution for cleavage of products from the resin and side chain deprotection:

   For each student, make 5 mL of this solution. We prepared these in individual vials for students to minimize transfer of the highly corrosive and hazardous solution.

Specific Notes:

Chloranil tests should yield blue/green beads following amine displacement reactions and colorless following acylation reactions to indicate reaction completion. Reactions that do not appear to have completed according to the test should be repeated.

Conditions for LC/MS analysis:

LC/MS analysis was carried out on a Dionex Ulitmate 3000 LC unit connected to a Thermo LCQ Fleet MS detector. Crude reaction mixtures were eluted from a Phenomenex Gemini C18 column (50 x 2 mm, 5 micron) using a flow rate of 0.5 mL/min (solvent A was 0.5% formic acid in water, and solvent B was 0.5% formic acid in methanol). Elution was effected using a linear gradient of 5-10% B for 5 min followed by a 2 min hold at 100% B, then column re-equilibration for 3 min. Peak elution was detected by UV (254 nm) and MS in positive ion mode.
